# Do Healthy Monarchs Migrate Farther? Tracking Natal Origins of Parasitized vs. Uninfected Monarch Butterflies Overwintering in Mexico

**DOI:** 10.1371/journal.pone.0141371

**Published:** 2015-11-25

**Authors:** Sonia Altizer, Keith A. Hobson, Andrew K. Davis, Jacobus C. De Roode, Leonard I. Wassenaar

**Affiliations:** 1 Odum School of Ecology, University of Georgia, Athens, Georgia, United States of America; 2 Environment Canada, 11 Innovation Blvd., Saskatoon, Saskatchewan, Canada; 3 Department of Biology, University of Western Ontario, London, Ontario, Canada; 4 Department of Biology, Emory University, Atlanta, Georgia, United States of America; Oxford Brookes University, UNITED KINGDOM

## Abstract

Long-distance migration can lower parasite prevalence if strenuous journeys remove infected animals from wild populations. We examined wild monarch butterflies (*Danaus plexippus*) to investigate the potential costs of the protozoan *Ophryocystis elektroscirrha* on migratory success. We collected monarchs from two wintering sites in central Mexico to compare infection status with hydrogen isotope (*δ* ^2^H) measurements as an indicator of latitude of origin at the start of fall migration. On average, uninfected monarchs had lower *δ* ^2^H values than parasitized butterflies, indicating that uninfected butterflies originated from more northerly latitudes and travelled farther distances to reach Mexico. Within the infected class, monarchs with higher quantitative spore loads originated from more southerly latitudes, indicating that heavily infected monarchs originating from farther north are less likely to reach Mexico. We ruled out the alternative explanation that lower latitudes give rise to more infected monarchs prior to the onset of migration using citizen science data to examine regional differences in parasite prevalence during the summer breeding season. We also found a positive association between monarch wing area and estimated distance flown. Collectively, these results emphasize that seasonal migrations can help lower infection levels in wild animal populations. Our findings, combined with recent declines in the numbers of migratory monarchs wintering in Mexico and observations of sedentary (winter breeding) monarch populations in the southern U.S., suggest that shifts from migratory to sedentary behavior will likely lead to greater infection prevalence for North American monarchs.

## Introduction

Many animals migrate long distances to follow seasonal changes in resources and suitable habitats [1,2]. For some species, these journeys span entire continents or hemispheres, can take several months to complete, and are accompanied by high energetic demands and extreme physiological changes [3,4]. Migration can have profound ecological and evolutionary consequences on a global scale [5], including effects on the spread and prevalence of infectious diseases [6]. For some species, migration increases pathogen exposure as animals move between and encounter different habitat types [7,8]. Long-distance migration can also have the opposite effect of reducing parasite prevalence (reviewed in [6]) if heavily infected animals migrate poorly (i.e., migratory culling), or if migration allows animals to escape from habitats where parasites have accumulated (i.e., migratory escape).

Interactions between monarch butterflies (*Danaus plexippus*) and a protozoan parasite (*Ophryocystis elektroscirrha*) have become a model system for studying the effects of seasonal migration on host-pathogen dynamics. Monarchs in eastern North America migrate up to 2500 km southwards each fall to discrete wintering sites in Central Mexico [9,10]. In spring, many of the same individuals migrate north to recolonize their breeding range [11,12]. Monarchs in western North America migrate shorter distances to winter along the coast of California [13], with increasing evidence that these monarchs intermix with eastern North American monarchs [14–16]. Monarchs also form non-migratory populations that breed year-round in locations such as southern Florida, Pacific and Caribbean Islands, and Central and South America [17,18].

The protozoan *O*. *elektroscirrha* (*OE*) is a specialist neogregarine sporozoan reported to infect monarch and queen (*D*. *gilippus*) butterflies [19,20]. Parasites are transmitted when infected adults scatter dormant spores onto milkweed leaves, especially during oviposition. Larvae ingest the spores, the parasites replicate within larval and pupal tissues, and butterflies emerge covered with millions of spores on the outsides of their bodies [19,20,21]. Previous research across multiple monarch populations suggested that parasite prevalence was lower in migratory compared to non-migratory populations [22], an effect that could stem from the combined effects of migratory culling and migratory escape. Infected monarchs were shown to have lower flight endurance and speed than healthy monarchs when flown in captivity [23]. Among monarchs in eastern North America, infection prevalence decreased as monarchs moved southwards during their fall migration, consistent with the prediction that infected monarchs migrate poorly [24]. However, more direct estimates of the relationship between parasite load and migration distance remain elusive, in part owing to difficulties in tracking migration success of healthy and infected butterflies in the field.

Here, we tested whether parasite infection predicts successful long-distance migration in eastern North American monarchs traveling different distances from natal to wintering grounds. We used stable-hydrogen isotope measurements of wings to link adults sampled at wintering sites in central Mexico to the natal grounds where they developed from larvae (following [25,26]). We hypothesized that the natal origins of parasitized monarchs that successfully reached Mexico would correspond more strongly to southerly latitudes (indicating that infected monarchs migrated shorter distances) compared to unparasitized butterflies, and we further hypothesized that estimated flight distances would decrease with increasing parasite loads within the subset of infected butterflies. We tested whether other factors (sex, wing area and measures of wing and body condition) predicted estimated migration distances, and used citizen science data from the previous summer to query whether variation in butterfly natal origins could be explained by more prevalent parasite infections in certain regions of eastern N. America.

## Methods

### Field sites and sampling wild monarchs

In February 2008, we collected adult monarchs at two discrete overwintering sites in the Neovolcanic Mountains of Central Mexico: (i) Sierra Chincua and (ii) Cerro Pelon. Both sites are located in Michoacan, Mexico [27,28]. Butterflies were collected using standard aerial nets with expandable poles. The prevalence of monarchs heavily infected with OE at each of these two locations in 2008 was 14.4 and 13.6%, respectively, based on a total sample of 1686 individuals. From ten replicate clusters (five clusters per site), we randomly selected healthy and infected butterflies following preliminary screening of parasite samples in the field, for a total of 75 healthy and 100 heavily infected monarchs. Monarchs were sampled under collecting permits from SEMARNAT (Secretaria de Medio Ambiente y Recursos Naturales, Permit # 08202) and the Monarch Biosphere Reserve (Mariposa Monarcha Reserva de la Biosfera, Permit # RBMM-DIRECT-0050.08), exported under permission from PROFEPA (Procuraduría Federal de Protección al Ambiente, Permit # 23811 21/Enero/2008) and imported with permission from the U.S. Fish and Wildlife Service and the US Department of Agriculture (P526P-06-02137).

### Measuring infection status

We initially used a non-destructive method to assess individual infection status by pressing clear adhesive stickers on adult abdomens (described in [22]). Spores of OE were counted at 50X magnification, and samples with >100 spores were considered to be heavily infected. This classification includes the two highest spore load categories as defined by Altizer et al. [22] and excludes the majority of monarchs that acquired spores through horizontal transfer among adults [29,30]. Monarchs with 0–20 spores per individual were considered in this analysis to be uninfected. After samples were returned to our laboratory, we obtained a quantitative measure of parasite infection for the subset of heavily infected monarchs. Monarch abdomens were vortexed at high speed in 5ml H_2_O for 5 mins and we estimated total spore loads per sample using a haemocytometer slide as described in De Roode et al. [21].

### Isotopic analysis and natal assignment

The right hind wing of each monarch was stored and processed for hydrogen isotope (*δ* ^2^H) analyses at the Stable Isotope Laboratory of Environment Canada, Saskatoon, Saskatchewan, Canada. Wings were placed in glass vials, solvent cleaned with a 2:1 chloroform:methanol solution to remove lipid residues and air dried. For *δ* ^2^H analysis, 0.35mg of wing tissue was loaded into 4.0 x 3.2 mm silver capsules. The Comparative Equilibration method to determine the *δ* ^2^H of non-exchangeable H in the wing tissue was used as described in Wassenaar and Hobson [26,31]. Lipid-free keratin standards (EC1—CBS, caribou hoof keratin, -197 ± 2 ‰; and EC2—KHS, kudu horn keratin, -54 ± 1 ‰) were used to normalize the results, and are reported in delta (*δ*) notation as parts per thousand (‰) deviation from the VSMOW–SLAP standard scale (Vienna Standard Mean Ocean Water–Standard Light Antarctic Precipitation).

The hydrogen isotopic analysis builds on previous work showing that, for the eastern breeding population of North America, *δ* ^2^H values in monarch wing chitin decrease linearly with increasing latitude [32]. In particular, the *δ* ^2^H in adult wing membranes is controlled by the geospatial location of the larval host plant, and associated isotopic values of precipitation [31, 32]. As a result, *δ* ^2^H has been used to estimate the latitudinal geographical origins of monarchs overwintering in Mexico [25], during the fall in Cuba [33], and during the spring recolonization in eastern N. America [12, 34]. The demonstrated regular pattern of depletion with latitude of *δ* ^2^H in wing chitin of known-origin monarchs throughout their eastern range [25], a phenomenon also demonstrated experimentally with monarchs raised on milkweed of known isotopic compositions [33], makes wing δ2H a convenient proxy for latitude in our study. This phenomenon is well known and apparent also in other taxa [35,36].

### Butterfly wing data

The dorsal sides of left and right monarch forewings were scanned with a flatbed scanner to obtain digital versions of their wings for measurement [37–40]. Using the FoveaPro plugin (Reindeer Graphics, Inc.) for Adobe Photoshop ®, we measured the area of each forewing in mm^2^. Forewing area is a predictor of among-population variation in migratory behavior [38], and therefore could impact individual flight distances. Prior to analysis, measures of left and right wings were averaged, and we excluded data for monarchs with torn or damaged forewings. Prior analyses examined monarch wing color in relation to monarch migratory status and flight propensity, and found that deeper orange hue was associated with migratory status and flight performance [37,38]. However, because the monarchs we examined here were over 5 months old and many had faded wings with visible scale loss, we chose not to include wing color in our analyses as the scale loss would affect color measurements.

### Citizen science data

To infer the geographic distribution of infection by *O*. *elektroscirrha* during the summer breeding period, we used Project Monarch Health (MH) data in which volunteers from across the US and Canada collected parasite samples from wild-caught monarchs by pressing transparent 1cm^2^ stickers against adult monarch abdomens. Samples were returned to the University of Georgia and scored for the presence/absence of heavy infection based on the presence of > 100 parasite spores per sample. Protocols for this program are described online (www.monarchparasites.org) and in Bartel et al. [24] and Satterfield et al. [41]. Across 2006–2011, a total of 124 volunteers participated from 23 states and two Canadian provinces. To make inferences about the relative contribution of different geographic regions to monarch infection, we focused on data for monarchs sampled between Aug 15 and Oct 15, 2007, as these monarchs would be most likely to have contributed to the overwintering population in Central Mexico sampled in Feb 2008. We recorded the latitude of each observer based on the city and state (or province) where monarchs were sampled, and further divided observer locations into one of three regions based on the following categories: South = between 30 to 36.9° latitude, N = 190; Central = between 37 to 41.49° latitude, N = 542; and North = between 41.5 to 49° latitude, N = 354. We omitted samples collected below 30° latitude, as monarchs have been reported to breed year-round at these sites, and thus are unlikely to contribute to the overwintering population in Mexico [41–43]. To limit observer-induced contamination from volunteer-derived MH samples, we removed data from observers for which prevalence was ≥ 60% based on five or more samples returned in a given year, as some observers in the early years of the project were less aware of the need to employ sterile rearing and sampling protocols, and thus unintentionally contaminated the majority of their samples. In total, we omitted 78 lines of data from 5 observers (out of an initial 1164 lines of data from 63 observers). One observer was omitted from the south, 3 observers were omitted from the central region, and 1 observer was omitted from the north.

### Data analysis

Analyses were conducted in SPSS ver. 19.0 [44]. We used logistic regression to examine the relationship between monarch infection status (assigned as 0/1 based whether or not monarchs were heavily infected or not) and the following dependent variables: *δ* ^2^H values (as a proxy for latitudinal origin and distance migrated), sex, site (Cerro Pelon or Sierra Chincua) and cluster (as a random variable nested within site). Next, we used general linear models (GLM) to test the relationship between infection status and natal origin in a different way, using *δ* ^2^H values as the dependent variable, and the following independent variables: infection status (assigned as 0/1 as before), site (Cerro Pelon or Sierra Chincua), cluster (as a random variable nested within site), sex, and forewing area. Using data for the subset of heavily infected monarchs only, we used linear regression to examine the relationship between *δ* ^2^H values (as the dependent variable) and quantitative spore load and forewing area (as independent variables).

For the analysis of regional differences in infection patterns from citizen science observations during summer 2007, we used logistic regression to examine the effect of latitude (as a continuous variable) on *OE* infection probability, treating each sample as an independent observation. We also used logistic regression to test for the effects of region (North, Central, South) on *OE* infection probability, with state nested within region as a separate variable to control for uneven sampling among states.

## Results

**Fig 1:**
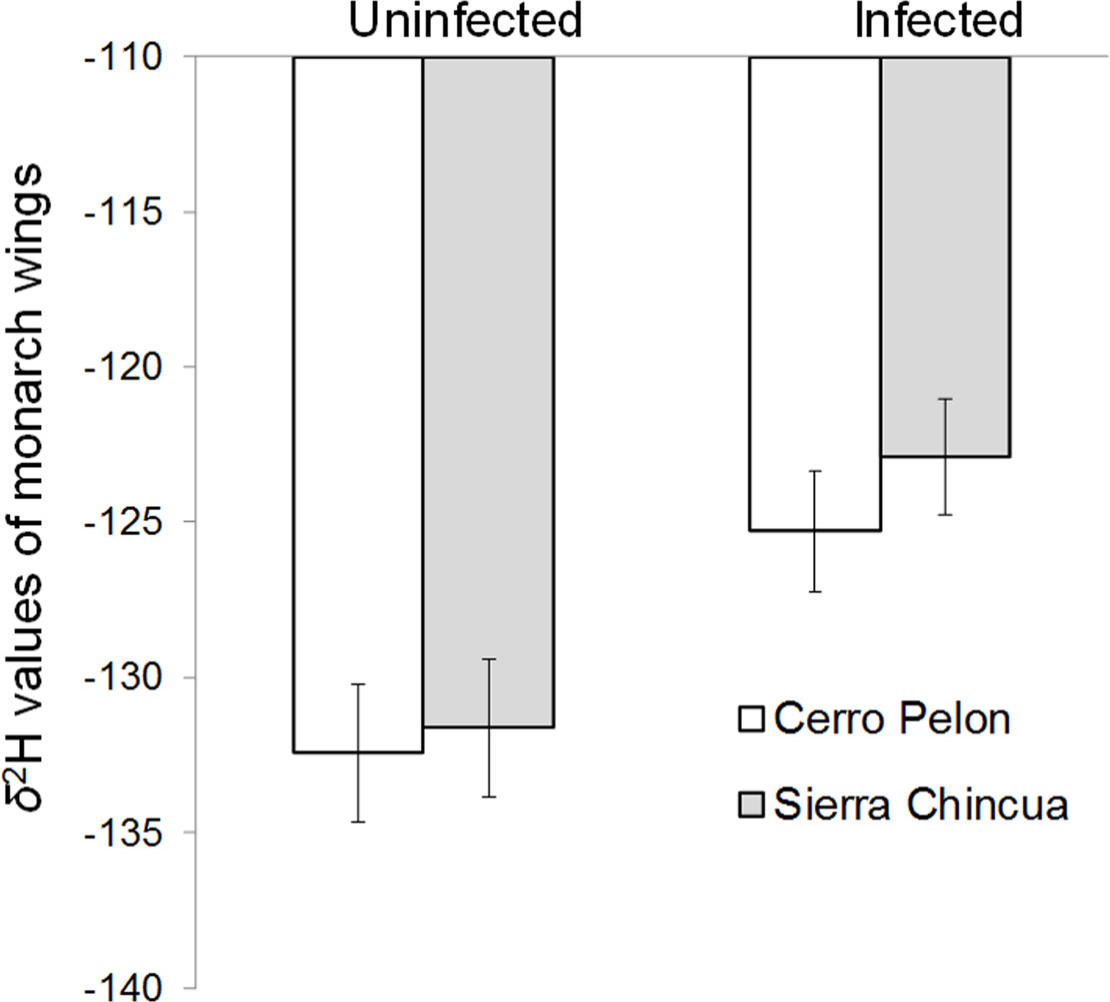
Average *δ* ^2^H values from wing membranes of overwintering butterflies in relation to monarch infection status (N = 75 uninfected and 100 infected monarchs). Lower *δ* ^2^H values correspond to more northerly latitudinal natal origins. Bars are colored according to two distinct overwintering sites in Central Mexico. Error bars show standard errors.

**Fig 2:**
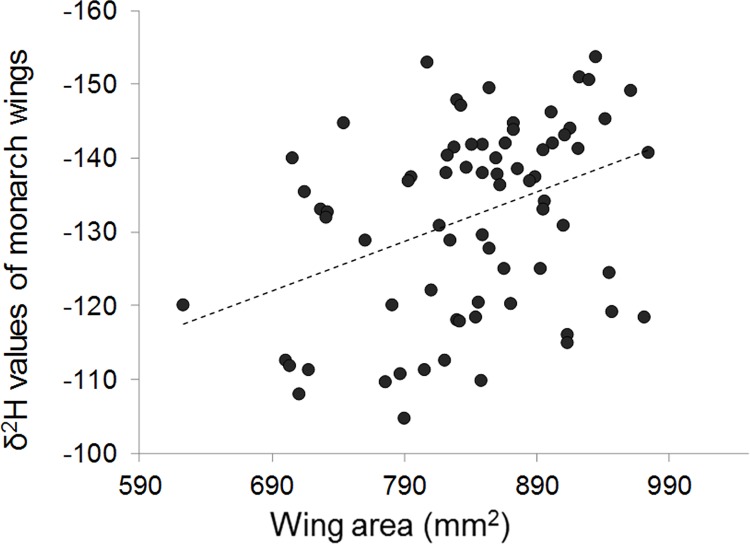
Monarch wing area in relation to *δ* ^2^H values. Lower *δ* ^2^H values correspond to more northerly latitudinal natal origins. Data shown for uninfected monarchs only, as the relationship was non-significant when tested using the subset of infected monarchs. Dashed line shows linear regression (β = -0.39, t = -3.53, P = 0.001; R^2^ = 0.15).

Logistic regression showed a strong positive relationship between infection probability and *δ* ^2^H as a proxy for north-south distance migrated, with infected monarchs, on average, originating from more southerly latitudes (Wald χ^2^ = 12.78, d.f. = 1, p < 0.0001; Fig 1). No other variables were significant predictors of monarch infection status (Sex: Wald χ2 = 0.451, d.f. = 1, p = 0.502; Colony: Wald χ2 = 0.019, d.f. = 1, p = 0.890; Cluster: Wald χ2 = 0.767, d.f. = 4, p = 0.943). GLM analysis of *δ* ^2^H values as the dependent variable showed that uninfected monarchs had significantly lower *δ* ^2^H values than heavily infected monarchs, again indicating uninfected individuals originated from farther north (F _1, 163_ = 13.43, p < 0.0001). This was true for monarchs sampled at the Sierra Chincua and Cerro Pelon overwintering sites, and we found no significant effects of colony, cluster, or sex on *δ* ^2^H values (colony: F _1, 163_ = 1.073, p = 0.333; cluster: F _6_, _163_ = 1.892, p = 0.085; sex: F _1, 163_ = 0.303, p = 0.583). Monarchs with larger forewings also had lower *δ* ^2^H values, indicating a positive relationship between the distance migrated and wing area (F _1, 163_ = 6.17; p = 0.014). This relationship between wing area and *δ* ^2^H was highly significant for the subset of uninfected monarchs (Fig 2) but was non-significant for infected monarchs.

Quantitative spore loads for heavily infected monarchs ranged from 5.01 x 10^3^ to 6.31 x 10^5^, representing a 125-fold difference in untransformed values. In a model focused on data for heavily infected monarchs only, we found a positive relationship between *δ* ^2^H values and log_10_-transformed spore load, indicating that monarchs with heavier parasite loads originated from more southerly latitudes (β = 5.63, d.f. = 99; t = 2.30, p = 0.023).

**Fig 3:**
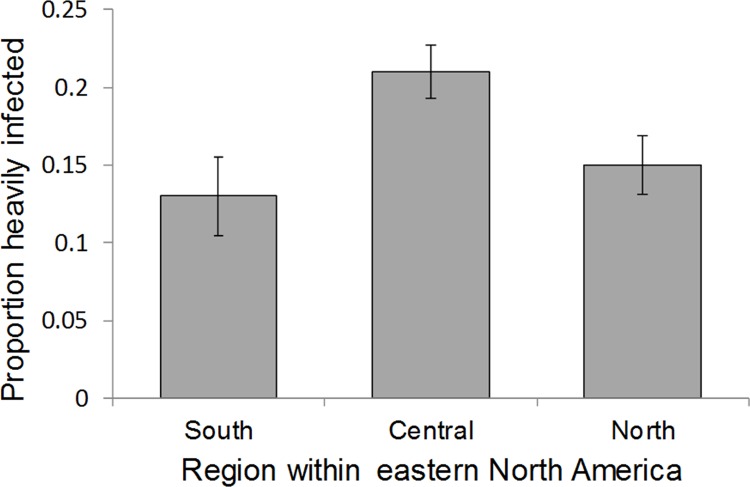
Proportion of monarchs heavily infected with *Ophryocystis* parasites based on citizen science monitoring in eastern North America during late summer and early fall 2007. Regions correspond to description in Methods as follows: South = between 30 to 36.9° latitude, Central = between 37 to 41.49° latitude, and North = between 41.5 to 49° latitude. Samples below 30°N latitude were excluded from analysis, and prevalence of infection was determined from Monarch Health data as described in Methods. Error bars represent standard errors.

Our analysis of infection patterns among the late summer / early fall 2007 monarchs sampled by volunteer observers showed no significant effect of latitude as a continuous variable on infection probability (Wald χ^2^ = 0.099, d.f. = 1, p = 0.753). When treated as a categorical variable, we found a higher infection probability among summer breeding monarchs sampled in the Central region relative to the North and South regions, but this effect was not significant (Wald χ^2^ = 0.000, d.f. = 2, p = 1.000; Fig 3) in an analysis that also included the effect of state nested within region (Wald χ^2^ = 18.10, d.f. = 17, p = 0.381; Fig 3). Including samples that were omitted from the 5 observers with high prevalence caused infection prevalence to increase to 19% for the North (N = 375), 23% for the Central region (N = 559) and 21% for the South (N = 230) relative to levels shown in Fig 3, but the effect of region on infection prevalence remained non-significant (Wald χ^2^ = 0.000, d.f. = 2, p = 1.000).

## Discussion

Our analysis showed that uninfected monarchs overwintering in Mexico travelled, on average, farther distances between their summer breeding and wintering sites than butterflies that were infected with the protozoan *O*. *elektroscirrha*. Moreover, within the class of infected hosts, monarchs with the heavier quantitative parasite loads originated from more southerly locations (closer to their wintering sites) compared to less heavily infected monarchs. One possible explanation for these findings is that heavily parasitized monarchs that originate from more northern latitudes (farthest from their wintering sites) simply do not reach Mexico, whereas healthy monarchs are better able to travel the farthest distances. This explanation would be consistent with the general idea that long-distance migration can lower pathogen prevalence by removing infected animals from the population (i.e., ‘migratory culling’, [6,23,24,45]). In this scenario, diseased animals suffering from infection are less likely to successfully migrate long distances owing to the combined physiological demands of migration and infection.

All evidence to date indicates that the fall migration and wintering period is energetically costly to monarchs, such that even a small cost of infection could be the tipping point between successful migration and premature death. Adult butterflies emerging in the late summer in eastern N. America weigh only ~0.5 g, yet must travel up to 5000 km round trip [46]. Moreover, monarchs use lipids stored during the fall to fuel their long distance flight and also to maintain themselves during the five-month overwintering period in Mexico, during which nectar resources are extremely limited [46,47]. Because OE infection causes reduced adult body size, shorter adult life span [21,48], and reduced flight performance in captive monarchs [23], the demands of migration likely remove a substantial fraction of heavily infected individuals each year, which would be consistent with the isotopic results reported here. Moreover, a previous field study showed that *O*. *elektroscirrha* prevalence decreased as monarchs moved southward along the east coast flyway during their annual fall migrations [24], consistent with the idea that infected animals migrate less successfully [45].

Evidence for hindered migratory ability among infected individuals has been reported for a few other animal species, including the fall armyworm moth (*Spodoptera frugiperda*) infected by an ectoparasitic nematode (*Noctuidonema guyanense*); in this case, adults appeared to have reduced migratory ability because few or no parasites were detected in moths recolonizing sites as they returned north [49]. Other work on Bewick’s swans (*Cygnus columbianus bewickii*) showed that infection by low-pathogenic avian influenza (LPAI) viruses delayed departure dates for fall migration by over a month, and reduced the travel distances of infected birds compared with healthy individuals [50]. Studies of other species showed little or no effect of infection state on migration [51–53] suggesting that some species can better tolerate infection during long distance journeys. This raises the possibility that migration could select for high infection tolerance, owing to the high fitness costs of migrating while harboring a debilitating pathogen.

We used citizen science data to examine an alternative explanation for the negative relationship observed between monarch infection status and latitude of natal origin (*δ* ^2^H) by testing whether lower latitudes late in the breeding season give rise to more infected monarchs. Although monarchs sampled in the central part of eastern N. America were more likely to be infected as compared to monarchs sampled in the north and south of their breeding range, this effect of region was not significant, and we further found no consistent effect of latitude as a continuous variable on late summer infection probability. It is also important to note that a recent analysis of OE infection probability across four separate years showed that regions with the highest prevalence differed among years [24], and in no years was late summer infection prevalence significantly higher at southern latitudes. Taken together, these results suggest that latitudinal differences in the natal origins of infected vs. uninfected monarchs that successfully reach Mexico are probably driven more by differences in migration ability than by differences in transmission among different breeding locations.

One possible confounding factor that could affect our analysis of quantitative spore loads within the infected class is that adult monarchs might lose spores from their bodies during the journey south, thus causing a negative relationship between quantitative spore load and distance migrated. In fact, an analysis of captive monarchs held in outdoor enclosures during the summer indicated that reproductively active adults could lose up to 90% of spores between eclosion and death [30]. Thus, although we found a five-fold difference in average spore load between monarchs at the extreme distributions of the *δ* ^2^H range, some portion of this difference could be due to the loss of spores during migration. On the other hand, direct comparison between the results of De Roode et al. [30] and our study are difficult because mating and oviposition in caged monarchs could lead to the loss of higher numbers of spores relative to flight alone (i.e., monarchs in the caged study were actively landing on plants and laying eggs continuously, whereas migrating monarchs spend a great deal of time in gliding flight). In addition, the loss of spores during migratory flight would not account for the differences in infection status as a binomial variable, because heavily infected butterflies would not lose enough spores to be misclassified as an uninfected monarch.

Our analysis also found a positive association between monarch wing area and estimated distance flown based on *δ* ^2^H. This same result was found in a prior (unpublished) investigation [54], and suggests that monarchs with larger wings are successful at migrating the farthest distances. While this conclusion seems intuitive, past evidence that wing size affects migration success (in monarchs) has only been circumstantial. For example, multiple studies showed that monarchs captured late in the fall migration season (which presumably fell behind) had smaller wings than those that migrated early [55–57]. This result could arise if monarchs with small wings fly more slowly and stay longer at stopover sites, resulting in a slower migration pace [58]. Slow-migrating monarchs are also at higher risk of mortality from extreme weather such as storms or falling temperatures [59]. In addition, long before the location of the overwintering sites were known, Beall [60] noted that monarchs found dead along the shore of Lake Erie were significantly smaller than those captured alive in this region during the fall, implying that small-winged monarchs are less successful at navigating water crossings within the flyway. Finally, monarchs from non-migratory populations tend to have smaller forewings than those from migratory populations [39,61]. Collectively, our results concerning monarch wing size are consistent with work on other migratory species showing that the demands of long-distance flight select for greater wing area to maximize powered and gliding flight [61–65].

We were surprised to find that the significant relationship between wing size and migration distance observed among uninfected monarchs did not appear in the infected group. We can only speculate as to the cause of this finding. It could be that the culling of smaller-winged monarchs is more likely over extremely long spans of travel, for monarchs that originate from the highest latitudes. Because the sample of infected monarchs successfully reaching Mexico is biased towards those that started out from more southerly origins, the culling of smaller monarchs might be less extreme at these shorter distances. In other words, it could be that infected monarchs that start out from more northerly locations simply do not reach Mexico, regardless of their wing size.

The monarchs’ fall migration in eastern North America is both unique and declining. Estimates of overwintering colony sizes fluctuate from year to year, but on average show evidence of a long-term decline of up to 90% during the past 20 years [66,67], with the last 3 consecutive years representing the lowest numbers of overwintering monarchs recorded in Mexico to date. The overall drop is thought to be due to loss of breeding habitat, especially within the Midwestern U.S. [68,69], which prior isotopic analysis identified as a major source of the Mexico overwintering population [25]. Over this same time period, some monarchs have become non-migratory and breed on exotic milkweed in the extreme southern US during the winter months rather than travel to Mexico [43,70]. A recent analysis of citizen science data on parasite infection showed that OE prevalence was markedly higher among winter breeding monarchs compared with migratory monarchs [41], suggesting that diminished migration increases infection risk. In combination with this recent work, results presented here predict that human activities that threaten monarch migration and cause shifts towards sedentary status may increase parasite transmission, potentially leading to greater population-wide infection prevalence across eastern North America.

In summary, our study is consistent with a growing body of scientific knowledge suggesting that infected migratory animals are less likely to successfully traverse long distances, and thereby highlights the role that migration can play in lowering parasitism in wild animal populations [6]. Thus, in contrast to human populations, for which long-distance travel can allow pathogens to spread across the globe in a matter of hours [71,72], migratory animals undertake strenuous long-distance journeys on their own power, and heavily infected animals might not survive these costly journeys. Importantly, monarch migrations, like the migrations of many other animal species [73], are considered an endangered phenomenon [74]. Already, human activities that discourage long-distance animal movements and encourage the formation of local year-round populations have enhanced the emergence of zoonotic pathogens in wildlife and humans [75,76]. Thus, results of our study underscore the need to conserve long-distance animal migrations to mitigate infection processes in wild animal populations, with implications for future disease risks in humans and threatened species.

## Supporting Information

S1 File Raw data used in analyses. Data are provided on isotopic values for field collected monarchs from two overwintering sites in central Mexico, February 2008, and on Monarch Health citizen science data tracking infection by the protozoan parasite *Ophryocystis elektroscirra* during late summer / early fall 2007.
(DOCX)
