# Supplementary material for: Do Healthy Monarchs Migrate Farther? Tracking Natal Origins of Parasitized vs. Uninfected Monarch Butterflies Overwintering in Mexico
Source: PLoS One. 2015 Nov 25;10(11):e0141371. doi: 10.1371/journal.pone.0141371 (PMC4659535; doi:10.1371/journal.pone.0141371)
Supplement: S1 File — Data are provided on isotopic values for field collected monarchs from two overwintering sites in central Mexico, February 2008, and on Monarch Health citizen science data tracking infection by the protozoan parasite Ophryocystis elektroscirra during late summer / early fall 2007. (DOCX) [file pone.0141371.s001.docx]

**Supporting Information**

Altizer et al. “Do healthy monarchs migrate farther? Tracking natal origins of parasitized vs. uninfected monarch butterflies overwintering in Mexico”

**S1 Table. Raw data on field collected monarchs from two overwintering sites in central Mexico, February 2008.** PercenH refers to % hydrogen in sample, samplewt refers to the weight of the wing membrane used to quantify δ^2^H, InfStatus refers to whether or not monarchs were heavily infected with the protozoan *Ophryocystis* *elektroscirrha*, Colony is the overwintering site name, Cluster is the specific aggregation of butterflies within a colony, WingArea refers to the total area of the forewing in mm^2^, LogSpore is the Log_10_ transformed number of parasite spores per infected monarch, and ForewingLength is the length of the forewing in mm.

| **IsoID** | **PercenH** | **SampleWt** | **FieldID** | **Delta2H** | **InfStatus** | **sex** | **Colony** | **Cluster** | **WingArea** | **LogSpore** | **ForewingLength** |
| --- | --- | --- | --- | --- | --- | --- | --- | --- | --- | --- | --- |
| G-89712 | 6.57 | 0.34 | 303 | -124.26 | 1 | F | Sierra Chincua | 2 | 884 | 4.18 | 56.24 |
| G-89714 | 6.23 | 0.34 | 306 | -140.3 | 1 | F | Sierra Chincua | 2 | 745 | 3.7 | 49.88 |
| G-76339 | 6.68 | 0.34 | 307 | -124.29 | 1 | M | Sierra Chincua | 2 | 897 | 5.03 | 54.32 |
| G-76259 | 6.4 | 0.36 | 311 | -139.99 | 0 | F | Sierra Chincua | 2 | 706 |  | 48.81 |
| G-89713 | 6.57 | 0.35 | 316 | -114.58 | 1 | F | Sierra Chincua | 2 | 930 | 3.88 | 54.79 |
| G-89733 | 6.46 | 0.33 | 325 | -120.21 | 1 | M | Sierra Chincua | 2 | 820 | 4.1 | 53.29 |
| G-76338 | 6.62 | 0.35 | 334 | -113.87 | 1 | M | Sierra Chincua | 2 | 870 | 5.48 | 53.5 |
| G-76271 | 6.7 | 0.34 | 358 | -147.81 | 0 | M | Sierra Chincua | 2 | 830 |  | 52.52 |
| G-76337 | 6.7 | 0.35 | 362 | -119.52 | 1 | F | Sierra Chincua | 2 | 843 | 4.88 | 52.45 |
| G-89732 | 6.5 | 0.35 | 404 | -136.97 | 1 | F | Sierra Chincua | 2 | 900 | 4.44 | 54.65 |
| G-76289 | 6.55 | 0.34 | 421 | -118.42 | 0 | M | Sierra Chincua | 2 | 971 |  | 56.54 |
| G-76288 | 6.55 | 0.36 | 422 | -112.66 | 0 | F | Sierra Chincua | 2 | 821 |  | 50.76 |
| G-76270 | 6.75 | 0.34 | 423 | -149.51 | 0 | F | Sierra Chincua | 2 | 854 |  | 51.87 |
| G-76269 | 6.67 | 0.35 | 424 | -149.11 | 0 | M | Sierra Chincua | 2 | 961 |  | 56.88 |
| G-76268 | 6.68 | 0.36 | 425 | -125.06 | 0 | F | Sierra Chincua | 2 | 866 |  | 53.41 |
| G-76267 | 6.98 | 0.34 | 426 | -144.77 | 0 | F | Sierra Chincua | 2 | 744 |  | 47.94 |
| G-76266 | 6.67 | 0.36 | 427 | -137.88 | 0 | F | Sierra Chincua | 2 | 861 |  | 53.05 |
| G-89659 | 6.37 | 0.35 | 428 | -109.61 | 0 | M | Sierra Chincua | 2 | 776 |  | 51.33 |
| G-89731 | 6.51 | 0.34 | 430 | -140.53 | 1 | F | Sierra Chincua | 2 | 808 | 4.4 | 50.23 |
| G-89730 | 6.41 | 0.33 | 433 | -111.14 | 1 | F | Sierra Chincua | 2 | 932 | 4.24 | 54.98 |
| G-89676 | 6.35 | 0.34 | 435 | -110.77 | 0 | F | Sierra Chincua | 2 | 787 |  | 51.59 |
| G-76336 | 6.66 | 0.36 | 444 | -104.74 | 1 | F | Sierra Chincua | 2 | 882 | 5.6 | 53.51 |
| G-76335 | 6.69 | 0.33 | 459 | -117.28 | 1 | F | Sierra Chincua | 2 | 825 | 5.15 | 51.12 |
| G-89729 | 6.49 | 0.33 | 473 | -110.08 | 1 | M | Sierra Chincua | 2 | 807 | 4.18 | 50.55 |
| G-76334 | 6.49 | 0.34 | 481 | -109.61 | 1 | M | Sierra Chincua | 2 | 836 | 5.58 | 52.09 |
| G-76333 | 6.68 | 0.35 | 489 | -125.29 | 1 | F | Sierra Chincua | 2 | 812 | 5.14 | 49.93 |
| G-76287 | 6.34 | 0.33 | 501 | -135.52 | 0 | F | Sierra Chincua | 3 | 715 |  | 48 |
| G-89728 | 6.56 | 0.35 | 502 | -132.72 | 1 | F | Sierra Chincua | 3 | 718 | 4.48 | 48.44 |
| G-89727 | 6.42 | 0.34 | 511 | -147.5 | 1 | F | Sierra Chincua | 3 | 852 | 4.45 | 53.02 |
| G-76286 | 6.74 | 0.34 | 512 | -133.04 | 0 | F | Sierra Chincua | 3 | 895 |  | 53.68 |
| G-89678 | 6.34 | 0.35 | 517 | -146.2 | 0 | F | Sierra Chincua | 3 | 901 |  | 53.11 |
| G-89679 | 6.24 | 0.34 | 527 | -127.81 | 0 | M | Sierra Chincua | 3 | 854 |  | 53.4 |
| G-89680 | 6.48 | 0.36 | 542 | -141.24 | 0 | M | Sierra Chincua | 3 | 922 |  | 55.64 |
| G-76332 | 6.68 | 0.36 | 545 | -120.42 | 1 | F | Sierra Chincua | 3 | 842 | 5.53 | 53.19 |
| G-89681 | 6.35 | 0.35 | 568 | -111.33 | 0 | F | Sierra Chincua | 3 | 806 |  | 50.9 |
| G-89726 | 6.6 | 0.33 | 585 | -128.05 | 1 | F | Sierra Chincua | 3 | 778 | 4.51 | 49.71 |
| G-76265 | 6.55 | 0.35 | 588 | -138.54 | 0 | M | Sierra Chincua | 3 | 876 |  | 53.67 |
| G-76264 | 6.71 | 0.34 | 589 | -138.08 | 0 | M | Sierra Chincua | 3 | 822 |  | 50.21 |
| G-89725 | 6.42 | 0.36 | 590 | -140.16 | 1 | F | Sierra Chincua | 3 | 814 | 3.88 | 51.25 |
| G-76331 | 6.59 | 0.37 | 607 | -129.31 | 1 | M | Sierra Chincua | 3 | 797 | 4.99 | 51.64 |
| G-76330 | 6.82 | 0.36 | 613 | -157.13 | 1 | F | Sierra Chincua | 3 | 914 | 4.93 | 56.12 |
| G-76329 | 6.49 | 0.36 | 619 | -139.55 | 1 | F | Sierra Chincua | 3 | 914 | 4.78 | 55.64 |
| G-76328 | 6.54 | 0.35 | 629 | -138.02 | 1 | F | Sierra Chincua | 3 | 901 | 4.74 | 53.3 |
| G-89724 | 6.41 | 0.34 | 664 | -99.8 | 1 | F | Sierra Chincua | 3 | 823 | 4.93 | 53.59 |
| G-76327 | 6.6 | 0.35 | 668 | -120.05 | 1 | F | Sierra Chincua | 3 | 848 | 5.84 | 53.56 |
| G-76326 | 6.57 | 0.36 | 678 | -119.39 | 1 | F | Sierra Chincua | 3 | 868 | 5.35 | 54.49 |
| G-89677 | 6.47 | 0.34 | 801 | -141.82 | 0 | M | Sierra Chincua | 4 | 849 |  | 53.05 |
| G-76285 | 6.49 | 0.36 | 802 | -137.96 | 0 | F | Sierra Chincua | 4 | 849 |  | 53.56 |
| G-76284 | 6.48 | 0.36 | 803 | -137.38 | 0 | F | Sierra Chincua | 4 | 795 |  | 51.11 |
| G-76283 | 6.57 | 0.36 | 817 | -140.07 | 0 | F | Sierra Chincua | 4 | 860 |  | 54.17 |
| G-76325 | 6.34 | 0.37 | 823 | -110.08 | 1 | F | Sierra Chincua | 4 | 873 | 5.01 | 53.79 |
| G-76282 | 6.63 | 0.35 | 830 | -144.81 | 0 | F | Sierra Chincua | 4 | 873 |  | 54.38 |
| G-89682 | 6.15 | 0.35 | 841 | -147.12 | 0 | M | Sierra Chincua | 4 | 833 |  | 52.5 |
| G-89722 | 6.4 | 0.34 | 854 | -117.34 | 1 | F | Sierra Chincua | 4 | 847 | 4.35 | 51.74 |
| G-76324 | 6.57 | 0.36 | 872 | -126.32 | 1 | F | Sierra Chincua | 4 | 793 | 5.08 | 51.45 |
| G-89723 | 6.46 | 0.33 | 882 | -113.1 | 1 | M | Sierra Chincua | 4 | 675 | 4.57 | 46.44 |
| G-89663 | 6.58 | 0.33 | 896 | -124.48 | 0 | M | Sierra Chincua | 4 | 945 |  | 56.2 |
| G-76323 | 6.76 | 0.37 | 900 | -135.37 | 1 | F | Sierra Chincua | 4 | 805 | 5.57 | 51.12 |
| G-89721 | 6.45 | 0.33 | 916 | -121.34 | 1 | M | Sierra Chincua | 4 | 955 | 4.3 | 54.42 |
| G-76322 | 6.6 | 0.33 | 959 | -137.24 | 1 | M | Sierra Chincua | 4 | 810 | 4.942 | 51.33 |
| G-76263 | 6.62 | 0.35 | 960 | -104.75 | 0 | F | Sierra Chincua | 4 | 790 |  | 51.43 |
| G-89720 | 6.34 | 0.34 | 971 | -138.76 | 1 | M | Sierra Chincua | 4 | 916 | 4.51 | 54.33 |
| G-76262 | 6.75 | 0.35 | 975 | -136.87 | 0 | M | Sierra Chincua | 4 | 885 |  | 53.17 |
| G-76281 | 6.56 | 0.34 | 987 | -144.02 | 0 | M | Sierra Chincua | 4 | 916 |  | 54.21 |
| G-76321 | 6.37 | 0.34 | 989 | -106.3 | 1 | F | Sierra Chincua | 4 | 782 | 5.16 | 51.23 |
| G-76280 | 6.42 | 0.37 | 996 | -116.03 | 0 | M | Sierra Chincua | 4 | 913 |  | 54.13 |
| G-76279 | 6.55 | 0.37 | 1008 | -109.92 | 0 | F | Sierra Chincua | 5 | 848 |  | 56.43 |
| G-76320 | 6.46 | 0.37 | 1009 | -135.37 | 1 | F | Sierra Chincua | 5 | 863 | 5.42 | 53.99 |
| G-76319 | 6.49 | 0.33 | 1018 | -131.66 | 1 | M | Sierra Chincua | 5 | 810 | 4.8 | 52.82 |
| G-76318 | 6.45 | 0.35 | 1020 | -109.89 | 1 | M | Sierra Chincua | 5 | 933 | 4.9 | 55.74 |
| G-76278 | 6.57 | 0.37 | 1022 | -112.55 | 0 | F | Sierra Chincua | 5 | 701 |  | 49.23 |
| G-76277 | 6.5 | 0.36 | 1029 | -145.29 | 0 | M | Sierra Chincua | 5 | 942 |  | 56.3 |
| G-89719 | 6.36 | 0.34 | 1031 | -128.9 | 1 | M | Sierra Chincua | 5 | 800 | 4.35 | 51.62 |
| G-76317 | 6.72 | 0.34 | 1033 | -103.01 | 1 | M | Sierra Chincua | 5 | 771 | 5.37 | 50.55 |
| G-76316 | 6.35 | 0.34 | 1034 | -108.09 | 1 | M | Sierra Chincua | 5 | 830 | 4.9 | 51.66 |
| G-76276 | 6.76 | 0.34 | 1036 | -133.16 | 0 | M | Sierra Chincua | 5 | 727 |  | 51.49 |
| G-76315 | 6.49 | 0.34 | 1082 | -113.43 | 1 | M | Sierra Chincua | 5 | 702 | 4.78 | 48.5 |
| G-76275 | 6.6 | 0.37 | 1087 | -130.94 | 0 | F | Sierra Chincua | 5 | 910 |  | 55.48 |
| G-76314 | 6.4 | 0.36 | 1088 | -91.86 | 1 | F | Sierra Chincua | 5 | 865 | 5.22 | 54.09 |
| G-76313 | 6.46 | 0.35 | 1096 | -112.32 | 1 | F | Sierra Chincua | 5 | 878 | 5.27 | 54.9 |
| G-76274 | 6.55 | 0.35 | 1097 | -108.03 | 0 | F | Sierra Chincua | 5 | 711 |  | 49.75 |
| G-89718 | 6.56 | 0.36 | 1118 | -137.94 | 1 | F | Sierra Chincua | 5 | 909 | 4.35 | 52.94 |
| G-76312 | 6.58 | 0.34 | 1128 | -127.06 | 1 | F | Sierra Chincua | 5 | 870 | 4.86 | 52.66 |
| G-76311 | 6.4 | 0.37 | 1130 | -108.05 | 1 | M | Sierra Chincua | 5 | 892 | 4.94 | 53.34 |
| G-76273 | 6.75 | 0.36 | 1133 | -120.29 | 0 | F | Sierra Chincua | 5 | 871 |  | 53.62 |
| G-89717 | 6.41 | 0.33 | 1140 | -117.04 | 1 | M | Sierra Chincua | 5 | 842 | 4.63 | 53 |
| G-76310 | 6.43 | 0.36 | 1149 | -110.73 | 1 | M | Sierra Chincua | 5 | 896 | 5.7 | 54.42 |
| G-76261 | 6.65 | 0.36 | 1154 | -143.22 | 0 | F | Sierra Chincua | 5 | 911 |  | 53.99 |
| G-76272 | 6.48 | 0.35 | 1162 | -137.48 | 0 | M | Sierra Chincua | 5 | 889 |  | 53.28 |
| G-89716 | 6.45 | 0.36 | 1164 | -123.47 | 1 | M | Sierra Chincua | 5 | 800 | 4 | 51.59 |
| G-76309 | 6.47 | 0.36 | 1182 | -145.33 | 1 | M | Sierra Chincua | 5 | 893 | 5.1 | 54.69 |
| G-76308 | 6.61 | 0.34 | 1187 | -134.2 | 1 | M | Sierra Chincua | 5 | 811 | 4.93 | 50.93 |
| G-89715 | 6.34 | 0.36 | 1191 | -108.5 | 1 | M | Sierra Chincua | 5 | 859 | 4.51 | 53.6 |
| G-76260 | 6.6 | 0.35 | 1201 | -111.23 | 0 | F | Cerro Pelon | 1 | 718 |  | 48.63 |
| G-89685 | 6.24 | 0.34 | 1202 | -152.74 | 1 | M | Cerro Pelon | 1 | 821 | 3.7 | 50.89 |
| G-76307 | 6.26 | 0.33 | 1222 | -132.77 | 1 | M | Cerro Pelon | 1 | 829 | 4.76 | 52 |
| G-76306 | 6.55 | 0.33 | 1241 | -117.4 | 1 | M | Cerro Pelon | 1 | 806 | 4.95 | 53.15 |
| G-76258 | 6.63 | 0.35 | 1263 | -134.24 | 0 | M | Cerro Pelon | 1 | 896 |  | 54.58 |
| G-89684 | 6.33 | 0.34 | 1266 | -142.12 | 0 | M | Cerro Pelon | 1 | 902 |  | 54.43 |
| G-76257 | 6.43 | 0.35 | 1275 | -128.8 | 0 | M | Cerro Pelon | 1 | 825 |  | 52.93 |
| G-89683 | 6.45 | 0.35 | 1277 | -154.43 | 1 | M | Cerro Pelon | 1 | 833 | 4.07 | 52.79 |
| G-89690 | 6.49 | 0.34 | 1281 | -119 | 1 | M | Cerro Pelon | 1 | 946 | 4.10 | 56.77 |
| G-76256 | 6.62 | 0.36 | 1284 | -136.43 | 0 | M | Cerro Pelon | 1 | 863 |  | 53.77 |
| G-89689 | 6.46 | 0.36 | 1292 | -117.63 | 1 | F | Cerro Pelon | 1 | 852 | 3.88 | 53.54 |
| G-89664 | 6.37 | 0.33 | 1313 | -140.83 | 0 | M | Cerro Pelon | 1 | 975 |  | 56.6 |
| G-89688 | 6.27 | 0.34 | 1336 | -134.65 | 1 | M | Cerro Pelon | 1 | 844 | 4.18 | 52.88 |
| G-89687 | 6.43 | 0.33 | 1339 | -126.09 | 1 | M | Cerro Pelon | 1 | 841 |  | 52.36 |
| G-89686 | 6.59 | 0.34 | 1352 | -133.46 | 1 | F | Cerro Pelon | 1 | 773 | 4.1 | 49.92 |
| G-89691 | 6.12 | 0.34 | 1361 | -101.08 | 1 | M | Cerro Pelon | 1 | 886 | 5.21 | 56.86 |
| G-89665 | 6.53 | 0.34 | 1365 | -129.58 | 0 | M | Cerro Pelon | 1 | 849 |  | 52.73 |
| G-76255 | 6.91 | 0.37 | 1400 | -150.98 | 0 | F | Cerro Pelon | 1 | 923 |  | 54.74 |
| G-89666 | 6.3 | 0.34 | 1526 | -153.09 | 0 | M | Cerro Pelon | 2 | 808 |  | 47.99 |
| G-89668 | 6.33 | 0.36 | 1527 | -132.69 | 0 | F | Cerro Pelon | 2 | 732 |  | 48.57 |
| G-89711 | 6.37 | 0.36 | 1531 | -133.54 | 1 | M | Cerro Pelon | 2 | 897 | 4.68 | 53.98 |
| G-76254 | 6.78 | 0.36 | 1549 | -125.06 | 0 | F | Cerro Pelon | 2 | 893 |  | 55.01 |
| G-89710 | 6.63 | 0.34 | 1574 | -141.68 | 1 | F | Cerro Pelon | 2 | 877 | 3.88 | 52.97 |
| G-76253 | 6.52 | 0.36 | 1579 | -122.04 | 0 | M | Cerro Pelon | 2 | 811 |  | 51.52 |
| G-76305 | 6.74 | 0.34 | 1585 | -145.49 | 1 | F | Cerro Pelon | 2 | 965 | 4.89 | 56.85 |
| G-89709 | 6.51 | 0.33 | 1627 | -147.2 | 1 | F | Cerro Pelon | 3 | 815 | 3.7 | 50.92 |
| G-76252 | 6.7 | 0.33 | 1628 | -130.86 | 0 | M | Cerro Pelon | 3 | 817 |  | 50.45 |
| G-76251 | 6.95 | 0.33 | 1629 | -131.93 | 0 | F | Cerro Pelon | 3 | 731 |  | 48.58 |
| G-89708 | 6.41 | 0.35 | 1630 | -111.78 | 1 | M | Cerro Pelon | 3 | 972 | 4.1 | 56.13 |
| G-89707 | 6.18 | 0.34 | 1638 | -127.41 | 1 | M | Cerro Pelon | 3 | 825 | 4.4 | 51.32 |
| G-76250 | 6.67 | 0.35 | 1639 | -138.66 | 0 | F | Cerro Pelon | 3 | 837 |  | 51.64 |
| G-89667 | 6.27 | 0.33 | 1667 | -120.43 | 0 | M | Cerro Pelon | 3 | 846 |  | 53 |
| G-76304 | 6.47 | 0.34 | 1677 | -136.61 | 1 | M | Cerro Pelon | 3 | 821 | 5.21 | 52.33 |
| G-89669 | 6.45 | 0.36 | 1689 | -150.54 | 0 | M | Cerro Pelon | 3 | 930 |  | 54.24 |
| G-76303 | 6.53 | 0.34 | 1690 | -135.19 | 1 | F | Cerro Pelon | 3 | 809 | 5.65 | 51.32 |
| G-89706 | 6.32 | 0.33 | 1693 | -124.08 | 1 | M | Cerro Pelon | 3 | 849 | 4.4 | 52.29 |
| G-89670 | 6.28 | 0.33 | 1694 | -114.94 | 0 | M | Cerro Pelon | 3 | 913 |  | 54.36 |
| G-76302 | 6.64 | 0.33 | 1699 | -121.76 | 1 | F | Cerro Pelon | 3 | 897 | 4.89 | 54.53 |
| G-76249 | 6.53 | 0.34 | 1700 | -141.82 | 0 | F | Cerro Pelon | 3 | 841 |  | 51.21 |
| G-89705 | 6.48 | 0.37 | 1716 | -134.12 | 1 | F | Cerro Pelon | 3 | 745 | 3.88 | 51.1 |
| G-76248 | 6.68 | 0.36 | 1717 | -117.8 | 0 | M | Cerro Pelon | 3 | 832 |  | 53.31 |
| G-76247 | 6.64 | 0.34 | 1719 | -118 | 0 | F | Cerro Pelon | 3 | 830 |  | 53.26 |
| G-89704 | 6.42 | 0.33 | 1720 | -132.42 | 1 | M | Cerro Pelon | 3 | 796 | 3.7 | 51.41 |
| G-76246 | 6.7 | 0.36 | 1726 | -143.93 | 0 | M | Cerro Pelon | 3 | 873 |  | 53.56 |
| G-76301 | 6.36 | 0.37 | 1727 | -111.15 | 1 | F | Cerro Pelon | 3 | 716 | 4.93 | 49.17 |
| G-76245 | 6.37 | 0.33 | 1729 | -136.87 | 0 | F | Cerro Pelon | 3 | 793 |  | 51.8 |
| G-76300 | 6.63 | 0.36 | 1736 | -132.02 | 1 | M | Cerro Pelon | 3 | 885 | 5.1 | 54.28 |
| G-89703 | 6.36 | 0.36 | 1741 | -120.41 | 1 | M | Cerro Pelon | 3 | 852 | 4.72 | 52.92 |
| G-89674 | 7.58 | 0.33 | 1742 | -160.2 | 0 | F | Cerro Pelon | 3 | 900 |  | 53.6 |
| G-89702 | 6.52 | 0.34 | 1746 | -135.63 | 1 | M | Cerro Pelon | 3 | 778 | 4.51 | 50.34 |
| G-76299 | 6.45 | 0.34 | 1751 | -130.65 | 1 | M | Cerro Pelon | 3 | 805 | 5.03 | 52.82 |
| G-89673 | 6.32 | 0.33 | 1752 | -153.81 | 0 | F | Cerro Pelon | 3 | 935 |  | 55.28 |
| G-89700 | 6.44 | 0.36 | 1769 | -113.26 | 1 | F | Cerro Pelon | 3 | 809 | 4.3 | 52.51 |
| G-76298 | 6.42 | 0.36 | 1774 | -126.18 | 1 | M | Cerro Pelon | 3 | 861 | 4.8 | 55.56 |
| G-89675 | 6.28 | 0.37 | 1775 | -118.49 | 0 | F | Cerro Pelon | 3 | 844 |  | 53.77 |
| G-76297 | 6.31 | 0.33 | 1789 | -106.41 | 1 | M | Cerro Pelon | 3 | 932 | 5.15 | 55.92 |
| G-89661 | 6.47 | 0.35 | 1790 | -140.34 | 0 | F | Cerro Pelon | 3 | 823 |  | 51.86 |
| G-89701 | 6.54 | 0.35 | 1803 | -105.68 | 1 | F | Cerro Pelon | 4 | 767 | 4.3 | 50.32 |
| G-89699 | 6.42 | 0.33 | 1804 | -125.45 | 1 | M | Cerro Pelon | 4 | 860 | 4.1 | 52.62 |
| G-89698 | 6.4 | 0.37 | 1830 | -111.42 | 1 | M | Cerro Pelon | 4 | 825 | 4.4 | 52.33 |
| G-89671 | 6.56 | 0.34 | 1832 | -141.99 | 0 | F | Cerro Pelon | 4 | 867 |  | 52.58 |
| G-89697 | 6.26 | 0.35 | 1833 | -104.95 | 1 | M | Cerro Pelon | 4 | 850 | 4 | 52.35 |
| G-89672 | 6.38 | 0.34 | 1835 | -117.58 | 0 | M | Cerro Pelon | 4 |  |  | 55.83 |
| G-76296 | 6.51 | 0.36 | 1860 | -117.72 | 1 | M | Cerro Pelon | 4 | 797 | 4.78 | 50.46 |
| G-76295 | 6.43 | 0.33 | 1878 | -131.3 | 1 | M | Cerro Pelon | 4 | 897 | 5.15 | 54.06 |
| G-89660 | 6.23 | 0.36 | 1883 | -119.18 | 0 | M | Cerro Pelon | 4 | 947 |  | 56.62 |
| G-76244 | 6.42 | 0.34 | 1887 | -111.77 | 0 | M | Cerro Pelon | 4 | 704 |  | 47.82 |
| G-76294 | 6.51 | 0.33 | 1888 | -108 | 1 | M | Cerro Pelon | 4 | 814 | 5.48 | 51.41 |
| G-89696 | 6.4 | 0.33 | 1911 | -111.44 | 1 | M | Cerro Pelon | 4 | 853 | 4.4 | 53.96 |
| G-89695 | 6.49 | 0.35 | 1912 | -129.16 | 1 | F | Cerro Pelon | 4 | 881 | 3.88 | 53.59 |
| G-76242 | 6.58 | 0.35 | 1913 | -141.42 | 0 | M | Cerro Pelon | 4 | 828 |  | 52.28 |
| G-89694 | 6.15 | 0.35 | 1916 | -106 | 1 | M | Cerro Pelon | 4 | 847 | 3.88 | 53.34 |
| G-76241 | 6.19 | 0.35 | 1917 | -120.14 | 0 | F | Cerro Pelon | 4 | 781 |  | 51.31 |
| G-76240 | 6.46 | 0.34 | 1922 | -141.11 | 0 | M | Cerro Pelon | 4 | 895 |  | 55.49 |
| G-76293 | 6.51 | 0.37 | 1930 | -127.79 | 1 | F | Cerro Pelon | 4 | 805 | 4.9 | 51.83 |
| G-76292 | 6.42 | 0.36 | 1932 | -109.24 | 1 | M | Cerro Pelon | 4 | 719 | 5.16 | 49.09 |
| G-76243 | 6.62 | 0.34 | 1933 | -120.16 | 0 | M | Cerro Pelon | 4 | 623 |  | 45.76 |
| G-89662 | 6.44 | 0.34 | 1973 | -128.89 | 0 | F | Cerro Pelon | 4 | 761 |  | 50.36 |
| G-76291 | 6.56 | 0.34 | 1978 | -132.41 | 1 | M | Cerro Pelon | 4 | 878 | 5.21 | 54.18 |
| G-89693 | 6.28 | 0.35 | 1983 | -115.12 | 1 | M | Cerro Pelon | 4 | 737 | 4.4 | 49.58 |
| G-76290 | 6.45 | 0.36 | 1987 | -137.57 | 1 | M | Cerro Pelon | 4 | 839 | 5.74 | 52.75 |
| G-89692 | 6.31 | 0.33 | 1989 | -135.1 | 1 | M | Cerro Pelon | 4 | 890 | 4.54 | 54.71 |

**S2 Table. Monarch Health citizen science data tracking infection by the protozoan *Ophryocystis elektroscirra*.** Data shown are for monarchs sampled by Monarch Health volunteers between Aug 15 and Oct 15, 2007. Latitude was assigned based on the city and state (or province) where monarchs were sampled, and we further divided observer locations into one of three regions based on the following categories: South = between 30 to 36.9 º latitude, N = 190; Central = between 37 to 41.49 º latitude, N = 542; and North = between 41.5 to 49 º latitude, N = 354. We omitted samples collected below 30 º latitude. InfYN refers to whether or not monarchs were heavily infected with the protozoan parasite based on examination of samples as described in the Methods section of the main text. The initials of each volunteer observer are provided here to maintain observer anonymity.

| **ID** | **Observer** | **State** | **Latitude** | **Region** | **InfYN** |
| --- | --- | --- | --- | --- | --- |
| 1285 | AB | OH | 39.6000 | Central | 0 |
| 1288 | AB | OH | 39.6000 | Central | 0 |
| 1289 | AB | OH | 39.6000 | Central | 0 |
| 1291 | AB | OH | 39.6000 | Central | 0 |
| 1287 | AB | OH | 39.6000 | Central | 0 |
| 1283 | AB | OH | 39.6000 | Central | 1 |
| 1284 | AB | OH | 39.6000 | Central | 0 |
| 1290 | AB | OH | 39.6000 | Central | 0 |
| 1286 | AB | OH | 39.6000 | Central | 1 |
| 1309 | AB | OH | 39.9000 | Central | 0 |
| 1312 | AB | OH | 39.9000 | Central | 0 |
| 1314 | AB | OH | 39.9000 | Central | 1 |
| 1308 | AB | OH | 39.9000 | Central | 0 |
| 1310 | AB | OH | 39.9000 | Central | 0 |
| 1311 | AB | OH | 39.9000 | Central | 0 |
| 1313 | AB | OH | 39.9000 | Central | 0 |
| 1316 | AB | OH | 39.9000 | Central | 0 |
| 1315 | AB | OH | 39.9000 | Central | 0 |
| 1317 | AB | OH | 39.9000 | Central | 1 |
| 1295 | AB | OH | 39.9500 | Central | 0 |
| 1296 | AB | OH | 39.9500 | Central | 0 |
| 1298 | AB | OH | 39.9500 | Central | 0 |
| 1306 | AB | OH | 39.9500 | Central | 0 |
| 1307 | AB | OH | 39.9500 | Central | 0 |
| 1293 | AB | OH | 39.9500 | Central | 0 |
| 1292 | AB | OH | 39.9500 | Central | 0 |
| 1294 | AB | OH | 39.9500 | Central | 0 |
| 1299 | AB | OH | 39.9500 | Central | 0 |
| 1300 | AB | OH | 39.9500 | Central | 0 |
| 1301 | AB | OH | 39.9500 | Central | 0 |
| 1302 | AB | OH | 39.9500 | Central | 0 |
| 1303 | AB | OH | 39.9500 | Central | 0 |
| 1297 | AB | OH | 39.9500 | Central | 0 |
| 1304 | AB | OH | 39.9500 | Central | 0 |
| 1305 | AB | OH | 39.9500 | Central | 1 |
| 2123 | AG | MN | 45.2700 | North | 0 |
| 2126 | AG | MN | 45.2700 | North | 0 |
| 2129 | AG | MN | 45.2700 | North | 0 |
| 2132 | AG | MN | 45.2700 | North | 0 |
| 2133 | AG | MN | 45.2700 | North | 0 |
| 2134 | AG | MN | 45.2700 | North | 0 |
| 2113 | AG | MN | 45.2700 | North | 0 |
| 2124 | AG | MN | 45.2700 | North | 0 |
| 2121 | AG | MN | 45.2700 | North | 0 |
| 2122 | AG | MN | 45.2700 | North | 0 |
| 2127 | AG | MN | 45.2700 | North | 0 |
| 2128 | AG | MN | 45.2700 | North | 0 |
| 2131 | AG | MN | 45.2700 | North | 0 |
| 2130 | AG | MN | 45.2700 | North | 1 |
| 2125 | AG | MN | 45.2700 | North | 0 |
| 2135 | AG | MN | 45.2700 | North | 0 |
| 1175 | AMG | SC | 34.3000 | South | 0 |
| 1169 | AMG | SC | 34.3000 | South | 1 |
| 1173 | AMG | SC | 34.3000 | South | 0 |
| 1163 | AMG | SC | 34.3000 | South | 0 |
| 1166 | AMG | SC | 34.3000 | South | 0 |
| 1170 | AMG | SC | 34.3000 | South | 0 |
| 1172 | AMG | SC | 34.3000 | South | 0 |
| 1177 | AMG | SC | 34.3000 | South | 1 |
| 1178 | AMG | SC | 34.3000 | South | 0 |
| 1165 | AMG | SC | 34.3000 | South | 0 |
| 1164 | AMG | SC | 34.3000 | South | 0 |
| 1168 | AMG | SC | 34.3000 | South | 0 |
| 1167 | AMG | SC | 34.3000 | South | 0 |
| 1171 | AMG | SC | 34.3000 | South | 0 |
| 1176 | AMG | SC | 34.3000 | South | 0 |
| 857 | BM | OH | 40.3000 | Central | 0 |
| 856 | BM | OH | 40.3000 | Central | 0 |
| 858 | BM | OH | 40.3000 | Central | 0 |
| 860 | BM | OH | 40.3000 | Central | 0 |
| 864 | BM | OH | 40.3000 | Central | 0 |
| 866 | BM | OH | 40.3000 | Central | 0 |
| 867 | BM | OH | 40.3000 | Central | 0 |
| 870 | BM | OH | 40.3000 | Central | 1 |
| 869 | BM | OH | 40.3000 | Central | 0 |
| 855 | BM | OH | 40.3000 | Central | 0 |
| 861 | BM | OH | 40.3000 | Central | 0 |
| 862 | BM | OH | 40.3000 | Central | 0 |
| 863 | BM | OH | 40.3000 | Central | 0 |
| 865 | BM | OH | 40.3000 | Central | 0 |
| 868 | BM | OH | 40.3000 | Central | 0 |
| 871 | BM | OH | 40.3000 | Central | 0 |
| 859 | BM | OH | 41.3500 | Central | 0 |
| 1557 | BP | ON, CANADA | 43.0000 | North | 0 |
| 1558 | BP | ON, CANADA | 43.0000 | North | 0 |
| 1559 | BP | ON, CANADA | 43.0000 | North | 0 |
| 1560 | BP | ON, CANADA | 43.0000 | North | 0 |
| 1562 | BP | ON, CANADA | 43.0000 | North | 0 |
| 1563 | BP | ON, CANADA | 43.0000 | North | 0 |
| 1565 | BP | ON, CANADA | 43.0000 | North | 0 |
| 1555 | BP | ON, CANADA | 43.0000 | North | 0 |
| 1556 | BP | ON, CANADA | 43.0000 | North | 0 |
| 1564 | BP | ON, CANADA | 43.0000 | North | 0 |
| 1566 | BP | ON, CANADA | 43.0000 | North | 0 |
| 1561 | BP | ON, CANADA | 43.0000 | North | 1 |
| 1212 | CS | MI | 41.9000 | North | 1 |
| 1213 | CS | MI | 41.9000 | North | 0 |
| 1218 | CS | MI | 41.9000 | North | 1 |
| 1221 | CS | MI | 41.9000 | North | 0 |
| 1222 | CS | MI | 41.9000 | North | 0 |
| 1223 | CS | MI | 41.9000 | North | 1 |
| 1225 | CS | MI | 41.9000 | North | 1 |
| 1231 | CS | MI | 41.9000 | North | 0 |
| 1233 | CS | MI | 41.9000 | North | 1 |
| 1235 | CS | MI | 41.9000 | North | 0 |
| 1236 | CS | MI | 41.9000 | North | 0 |
| 1238 | CS | MI | 41.9000 | North | 0 |
| 1214 | CS | MI | 41.9000 | North | 0 |
| 1215 | CS | MI | 41.9000 | North | 0 |
| 1216 | CS | MI | 41.9000 | North | 1 |
| 1217 | CS | MI | 41.9000 | North | 0 |
| 1220 | CS | MI | 41.9000 | North | 0 |
| 1224 | CS | MI | 41.9000 | North | 0 |
| 1219 | CS | MI | 41.9000 | North | 1 |
| 1226 | CS | MI | 41.9000 | North | 0 |
| 1227 | CS | MI | 41.9000 | North | 0 |
| 1228 | CS | MI | 41.9000 | North | 0 |
| 1229 | CS | MI | 41.9000 | North | 0 |
| 1230 | CS | MI | 41.9000 | North | 0 |
| 1232 | CS | MI | 41.9000 | North | 0 |
| 1234 | CS | MI | 41.9000 | North | 0 |
| 1237 | CS | MI | 41.9000 | North | 1 |
| 877 | CG | MB | 49.0000 | North | 1 |
| 875 | CG | MB | 49.0000 | North | 0 |
| 872 | CG | MB | 49.0000 | North | 0 |
| 876 | CG | MB | 49.0000 | North | 0 |
| 873 | CG | MB | 49.0000 | North | 0 |
| 874 | CG | MB | 49.0000 | North | 0 |
| 2082 | CR | GA | 34.0000 | South | 0 |
| 2087 | CR | GA | 34.0000 | South | 0 |
| 2084 | CR | GA | 34.0000 | South | 0 |
| 2088 | CR | GA | 34.0000 | South | 0 |
| 2078 | CR | GA | 34.0000 | South | 0 |
| 2085 | CR | GA | 34.0000 | South | 0 |
| 2081 | CR | GA | 34.0000 | South | 1 |
| 2079 | CR | GA | 34.0000 | South | 1 |
| 2083 | CR | GA | 34.0000 | South | 0 |
| 2080 | CR | GA | 34.0000 | South | 0 |
| 2086 | CR | GA | 34.0000 | South | 0 |
| 1538 | CJ | MN | 44.2000 | North | 0 |
| 1534 | CJ | MN | 44.2000 | North | 0 |
| 1527 | CJ | MN | 44.2000 | North | 0 |
| 1541 | CJ | MN | 44.2000 | North | 0 |
| 1545 | CJ | MN | 44.2000 | North | 0 |
| 1535 | CJ | MN | 44.2000 | North | 0 |
| 1536 | CJ | MN | 44.2000 | North | 0 |
| 1537 | CJ | MN | 44.2000 | North | 0 |
| 1539 | CJ | MN | 44.2000 | North | 0 |
| 1542 | CJ | MN | 44.2000 | North | 0 |
| 1543 | CJ | MN | 44.2000 | North | 0 |
| 1544 | CJ | MN | 44.2000 | North | 0 |
| 1540 | CJ | MN | 44.2000 | North | 1 |
| 1546 | CJ | MN | 44.2000 | North | 0 |
| 1650 | DT | OH | 41.6000 | North | 0 |
| 1653 | DT | OH | 41.6000 | North | 0 |
| 1654 | DT | OH | 41.6000 | North | 0 |
| 1655 | DT | OH | 41.6000 | North | 0 |
| 1656 | DT | OH | 41.6000 | North | 0 |
| 1657 | DT | OH | 41.6000 | North | 0 |
| 1651 | DT | OH | 41.6000 | North | 0 |
| 1652 | DT | OH | 41.6000 | North | 1 |
| 1071 | DC | OH | 39.0000 | Central | 0 |
| 1072 | DC | OH | 39.3000 | Central | 0 |
| 1073 | DC | OH | 39.3000 | Central | 0 |
| 1742 | DL | OH | 39.9500 | Central | 0 |
| 1743 | DL | OH | 39.9500 | Central | 0 |
| 1746 | DL | OH | 39.9500 | Central | 0 |
| 1749 | DL | OH | 39.9500 | Central | 0 |
| 1753 | DL | OH | 39.9500 | Central | 0 |
| 1754 | DL | OH | 39.9500 | Central | 0 |
| 1755 | DL | OH | 39.9500 | Central | 0 |
| 1741 | DL | OH | 39.9500 | Central | 0 |
| 1744 | DL | OH | 39.9500 | Central | 0 |
| 1745 | DL | OH | 39.9500 | Central | 0 |
| 1747 | DL | OH | 39.9500 | Central | 0 |
| 1748 | DL | OH | 39.9500 | Central | 0 |
| 1750 | DL | OH | 39.9500 | Central | 0 |
| 1751 | DL | OH | 39.9500 | Central | 0 |
| 1752 | DL | OH | 39.9500 | Central | 0 |
| 1756 | DL | OH | 39.9500 | Central | 0 |
| 1209 | EK | SC | 34.8000 | South | 0 |
| 1210 | EK | SC | 34.8000 | South | 0 |
| 1208 | EK | SC | 34.8000 | South | 0 |
| 1207 | EK | SC | 34.8000 | South | 0 |
| 1211 | EK | SC | 34.8000 | South | 0 |
| 1333 | GJ | MA | 41.6300 | North | 0 |
| 1012 | IM | GA | 34.0000 | South | 0 |
| 1958 | IG | MI | 41.9000 | North | 0 |
| 1960 | IG | MI | 41.9000 | North | 0 |
| 1947 | IG | MI | 41.9000 | North | 0 |
| 1945 | IG | MI | 41.9000 | North | 1 |
| 1953 | IG | MI | 41.9000 | North | 0 |
| 1954 | IG | MI | 41.9000 | North | 1 |
| 1956 | IG | MI | 41.9000 | North | 0 |
| 1955 | IG | MI | 41.9000 | North | 1 |
| 1948 | IG | MI | 41.9000 | North | 0 |
| 1950 | IG | MI | 41.9000 | North | 0 |
| 1944 | IG | MI | 41.9000 | North | 1 |
| 1946 | IG | MI | 41.9000 | North | 1 |
| 1957 | IG | MI | 41.9000 | North | 0 |
| 1951 | IG | MI | 41.9000 | North | 0 |
| 1952 | IG | MI | 41.9000 | North | 1 |
| 1949 | IG | MI | 41.9000 | North | 1 |
| 1959 | IG | MI | 41.9000 | North | 0 |
| 1678 | JA | MI | 41.9000 | North | 0 |
| 1676 | JA | MI | 41.9000 | North | 1 |
| 1677 | JA | MI | 41.9000 | North | 0 |
| 1679 | JA | MI | 41.9000 | North | 0 |
| 1680 | JA | MI | 41.9000 | North | 0 |
| 1681 | JA | MI | 41.9000 | North | 0 |
| 1682 | JA | MI | 41.9000 | North | 0 |
| 1683 | JA | MI | 41.9000 | North | 0 |
| 1684 | JA | MI | 41.9000 | North | 0 |
| 1670 | JA | IA | 42.0000 | North | 0 |
| 1671 | JA | IA | 42.0000 | North | 0 |
| 1672 | JA | IA | 42.0000 | North | 0 |
| 1673 | JA | IA | 42.0000 | North | 0 |
| 1675 | JA | IA | 42.0000 | North | 0 |
| 1674 | JA | IA | 42.0000 | North | 1 |
| 1692 | JH | IA | 41.5000 | North | 1 |
| 1697 | JH | IA | 41.5000 | North | 0 |
| 1700 | JH | IA | 41.5000 | North | 0 |
| 1701 | JH | IA | 41.5000 | North | 0 |
| 1691 | JH | IA | 41.5000 | North | 0 |
| 1696 | JH | IA | 41.5000 | North | 0 |
| 1698 | JH | IA | 41.5000 | North | 0 |
| 1693 | JH | IA | 41.5000 | North | 0 |
| 1699 | JH | IA | 41.5000 | North | 0 |
| 1694 | JH | IA | 41.5000 | North | 1 |
| 1695 | JH | IA | 41.5000 | North | 1 |
| 1702 | JH | IA | 41.5000 | North | 1 |
| 1016 | JA | GA | 33.5000 | South | 0 |
| 1014 | JA | GA | 33.5000 | South | 0 |
| 1015 | JA | GA | 33.5000 | South | 0 |
| 1757 | JR | MI | 41.9000 | North | 0 |
| 1758 | JR | MI | 41.9000 | North | 0 |
| 1759 | JR | MI | 41.9000 | North | 0 |
| 1669 | JR | MN | 43.7000 | North | 0 |
| 853 | JK | WI | 42.7000 | North | 0 |
| 854 | JK | WI | 43.9000 | North | 0 |
| 1066 | JM | FL | 29.6500 | South | 0 |
| 1070 | JM | FL | 29.6500 | South | 1 |
| 1068 | JM | FL | 29.6500 | South | 0 |
| 1067 | JM | GA | 33.7000 | South | 0 |
| 1069 | JM | GA | 33.7000 | South | 0 |
| 1065 | JM | GA | 33.7000 | South | 0 |
| 983 | JME | NC | 36.5000 | South | 1 |
| 984 | JME | NC | 36.5000 | South | 1 |
| 987 | JME | NC | 36.5000 | South | 0 |
| 988 | JME | NC | 36.5000 | South | 0 |
| 998 | JME | NC | 36.5000 | South | 1 |
| 992 | JME | NC | 36.5000 | South | 0 |
| 995 | JME | NC | 36.5000 | South | 0 |
| 991 | JME | NC | 36.5000 | South | 0 |
| 990 | JME | NC | 36.5000 | South | 0 |
| 993 | JME | NC | 36.5000 | South | 0 |
| 982 | JME | NC | 36.5000 | South | 0 |
| 981 | JME | NC | 36.5000 | South | 0 |
| 985 | JME | NC | 36.5000 | South | 0 |
| 986 | JME | NC | 36.5000 | South | 0 |
| 989 | JME | NC | 36.5000 | South | 1 |
| 997 | JME | NC | 36.5000 | South | 1 |
| 994 | JME | NC | 36.5000 | South | 0 |
| 996 | JME | NC | 36.5000 | South | 0 |
| 610 | JS | MI | 41.9000 | North | 0 |
| 1133 | JS | MI | 41.9000 | North | 0 |
| 1131 | JS | MI | 41.9000 | North | 0 |
| 1136 | JS | MI | 41.9000 | North | 0 |
| 1138 | JS | MI | 41.9000 | North | 0 |
| 613 | JS | MI | 41.9000 | North | 0 |
| 1132 | JS | MI | 41.9000 | North | 0 |
| 1134 | JS | MI | 41.9000 | North | 0 |
| 1135 | JS | MI | 41.9000 | North | 0 |
| 1137 | JS | MI | 41.9000 | North | 0 |
| 1139 | JS | MI | 41.9000 | North | 0 |
| 620 | JS | MI | 41.9000 | North | 0 |
| 617 | JS | MI | 41.9000 | North | 0 |
| 612 | JS | MI | 41.9000 | North | 0 |
| 774 | KB | MI | 41.9000 | North | 0 |
| 775 | KB | MI | 41.9000 | North | 0 |
| 776 | KB | MI | 41.9000 | North | 0 |
| 778 | KB | MI | 41.9000 | North | 0 |
| 781 | KB | MI | 41.9000 | North | 0 |
| 782 | KB | MI | 41.9000 | North | 0 |
| 783 | KB | MI | 41.9000 | North | 0 |
| 785 | KB | MI | 41.9000 | North | 0 |
| 786 | KB | MI | 41.9000 | North | 0 |
| 777 | KB | MI | 41.9000 | North | 0 |
| 779 | KB | MI | 41.9000 | North | 0 |
| 780 | KB | MI | 41.9000 | North | 0 |
| 784 | KB | MI | 41.9000 | North | 0 |
| 748 | KK | MI | 41.9000 | North | 0 |
| 1855 | KS | NC | 35.8000 | South | 0 |
| 1856 | KS | NC | 35.8000 | South | 0 |
| 1857 | KS | NC | 35.8000 | South | 0 |
| 1859 | KS | NC | 35.8000 | South | 0 |
| 1860 | KS | NC | 35.8000 | South | 0 |
| 1861 | KS | NC | 35.8000 | South | 0 |
| 1862 | KS | NC | 35.8000 | South | 0 |
| 1863 | KS | NC | 35.8000 | South | 0 |
| 1864 | KS | NC | 35.8000 | South | 0 |
| 1858 | KS | NC | 35.8000 | South | 0 |
| 894 | LB | MN | 47.7700 | North | 1 |
| 907 | LB | MN | 47.7700 | North | 0 |
| 897 | LB | MN | 47.7700 | North | 0 |
| 905 | LB | MN | 47.7700 | North | 0 |
| 902 | LB | MN | 47.7700 | North | 0 |
| 901 | LB | MN | 47.7700 | North | 0 |
| 892 | LB | MN | 47.7700 | North | 1 |
| 896 | LB | MN | 47.7700 | North | 1 |
| 895 | LB | MN | 47.7700 | North | 1 |
| 898 | LB | MN | 47.7700 | North | 0 |
| 899 | LB | MN | 47.7700 | North | 0 |
| 906 | LB | MN | 47.7700 | North | 0 |
| 904 | LB | MN | 47.7700 | North | 1 |
| 903 | LB | MN | 47.7700 | North | 1 |
| 900 | LB | MN | 47.7700 | North | 0 |
| 893 | LB | MN | 47.7700 | North | 1 |
| 908 | LB | MN | 47.7700 | North | 0 |
| 909 | LB | MN | 47.7700 | North | 1 |
| 1526 | LW | NC | 35.7000 | South | 0 |
| 1013 | LBR | GA | 33.7000 | South | 0 |
| 1319 | LGB | GA | 32.4000 | South | 0 |
| 1318 | LGB | GA | 33.9000 | South | 0 |
| 1011 | ML | GA | 34.1000 | South | 0 |
| 1422 | MV | NJ | 39.9800 | Central | 0 |
| 1423 | MV | NJ | 39.9800 | Central | 0 |
| 1427 | MV | NJ | 39.9800 | Central | 0 |
| 1428 | MV | NJ | 39.9800 | Central | 0 |
| 1430 | MV | NJ | 39.9800 | Central | 0 |
| 1432 | MV | NJ | 39.9800 | Central | 0 |
| 1435 | MV | NJ | 39.9800 | Central | 0 |
| 1437 | MV | NJ | 39.9800 | Central | 0 |
| 1440 | MV | NJ | 39.9800 | Central | 0 |
| 1441 | MV | NJ | 39.9800 | Central | 0 |
| 1445 | MV | NJ | 39.9800 | Central | 0 |
| 1455 | MV | NJ | 39.9800 | Central | 0 |
| 1456 | MV | NJ | 39.9800 | Central | 0 |
| 1457 | MV | NJ | 39.9800 | Central | 0 |
| 1459 | MV | NJ | 39.9800 | Central | 0 |
| 1460 | MV | NJ | 39.9800 | Central | 0 |
| 1461 | MV | NJ | 39.9800 | Central | 0 |
| 1463 | MV | NJ | 39.9800 | Central | 0 |
| 1464 | MV | NJ | 39.9800 | Central | 0 |
| 1466 | MV | NJ | 39.9800 | Central | 0 |
| 1470 | MV | NJ | 39.9800 | Central | 0 |
| 1467 | MV | NJ | 39.9800 | Central | 0 |
| 1471 | MV | NJ | 39.9800 | Central | 0 |
| 1472 | MV | NJ | 39.9800 | Central | 0 |
| 1473 | MV | NJ | 39.9800 | Central | 0 |
| 1474 | MV | NJ | 39.9800 | Central | 0 |
| 1475 | MV | NJ | 39.9800 | Central | 0 |
| 1476 | MV | NJ | 39.9800 | Central | 0 |
| 1480 | MV | NJ | 39.9800 | Central | 0 |
| 1484 | MV | NJ | 39.9800 | Central | 0 |
| 1485 | MV | NJ | 39.9800 | Central | 0 |
| 1486 | MV | NJ | 39.9800 | Central | 0 |
| 1487 | MV | NJ | 39.9800 | Central | 0 |
| 1488 | MV | NJ | 39.9800 | Central | 0 |
| 1489 | MV | NJ | 39.9800 | Central | 0 |
| 1500 | MV | NJ | 39.9800 | Central | 0 |
| 1501 | MV | NJ | 39.9800 | Central | 0 |
| 1504 | MV | NJ | 39.9800 | Central | 0 |
| 1505 | MV | NJ | 39.9800 | Central | 0 |
| 1508 | MV | NJ | 39.9800 | Central | 0 |
| 1509 | MV | NJ | 39.9800 | Central | 0 |
| 1511 | MV | NJ | 39.9800 | Central | 0 |
| 1512 | MV | NJ | 39.9800 | Central | 0 |
| 1515 | MV | NJ | 39.9800 | Central | 0 |
| 1516 | MV | NJ | 39.9800 | Central | 0 |
| 1517 | MV | NJ | 39.9800 | Central | 1 |
| 1520 | MV | NJ | 39.9800 | Central | 0 |
| 1521 | MV | NJ | 39.9800 | Central | 0 |
| 1525 | MV | NJ | 39.9800 | Central | 0 |
| 1524 | MV | NJ | 39.9800 | Central | 1 |
| 1425 | MV | NJ | 39.9800 | Central | 0 |
| 1424 | MV | NJ | 39.9800 | Central | 0 |
| 1431 | MV | NJ | 39.9800 | Central | 0 |
| 1429 | MV | NJ | 39.9800 | Central | 0 |
| 1433 | MV | NJ | 39.9800 | Central | 0 |
| 1434 | MV | NJ | 39.9800 | Central | 0 |
| 1436 | MV | NJ | 39.9800 | Central | 0 |
| 1438 | MV | NJ | 39.9800 | Central | 0 |
| 1439 | MV | NJ | 39.9800 | Central | 0 |
| 1442 | MV | NJ | 39.9800 | Central | 0 |
| 1443 | MV | NJ | 39.9800 | Central | 0 |
| 1444 | MV | NJ | 39.9800 | Central | 0 |
| 1446 | MV | NJ | 39.9800 | Central | 0 |
| 1447 | MV | NJ | 39.9800 | Central | 0 |
| 1448 | MV | NJ | 39.9800 | Central | 0 |
| 1449 | MV | NJ | 39.9800 | Central | 0 |
| 1450 | MV | NJ | 39.9800 | Central | 0 |
| 1451 | MV | NJ | 39.9800 | Central | 0 |
| 1452 | MV | NJ | 39.9800 | Central | 0 |
| 1453 | MV | NJ | 39.9800 | Central | 0 |
| 1454 | MV | NJ | 39.9800 | Central | 0 |
| 1458 | MV | NJ | 39.9800 | Central | 0 |
| 1462 | MV | NJ | 39.9800 | Central | 0 |
| 1468 | MV | NJ | 39.9800 | Central | 0 |
| 1469 | MV | NJ | 39.9800 | Central | 0 |
| 1465 | MV | NJ | 39.9800 | Central | 1 |
| 1477 | MV | NJ | 39.9800 | Central | 0 |
| 1478 | MV | NJ | 39.9800 | Central | 0 |
| 1479 | MV | NJ | 39.9800 | Central | 0 |
| 1481 | MV | NJ | 39.9800 | Central | 0 |
| 1482 | MV | NJ | 39.9800 | Central | 1 |
| 1483 | MV | NJ | 39.9800 | Central | 0 |
| 1490 | MV | NJ | 39.9800 | Central | 0 |
| 1491 | MV | NJ | 39.9800 | Central | 0 |
| 1492 | MV | NJ | 39.9800 | Central | 0 |
| 1493 | MV | NJ | 39.9800 | Central | 0 |
| 1494 | MV | NJ | 39.9800 | Central | 0 |
| 1495 | MV | NJ | 39.9800 | Central | 0 |
| 1496 | MV | NJ | 39.9800 | Central | 0 |
| 1497 | MV | NJ | 39.9800 | Central | 0 |
| 1498 | MV | NJ | 39.9800 | Central | 0 |
| 1499 | MV | NJ | 39.9800 | Central | 0 |
| 1506 | MV | NJ | 39.9800 | Central | 0 |
| 1507 | MV | NJ | 39.9800 | Central | 0 |
| 1514 | MV | NJ | 39.9800 | Central | 0 |
| 1503 | MV | NJ | 39.9800 | Central | 0 |
| 1513 | MV | NJ | 39.9800 | Central | 0 |
| 1510 | MV | NJ | 39.9800 | Central | 0 |
| 1502 | MV | NJ | 39.9800 | Central | 1 |
| 1518 | MV | NJ | 39.9800 | Central | 0 |
| 1519 | MV | NJ | 39.9800 | Central | 1 |
| 1522 | MV | NJ | 39.9800 | Central | 0 |
| 1523 | MV | NJ | 39.9800 | Central | 1 |
| 1819 | MB | GA | 34.3000 | South | 0 |
| 1820 | MB | GA | 34.3000 | South | 0 |
| 1822 | MB | GA | 34.3000 | South | 0 |
| 1821 | MB | GA | 34.3000 | South | 0 |
| 1818 | MB | GA | 34.3000 | South | 1 |
| 1823 | MB | GA | 34.3000 | South | 0 |
| 1824 | MB | GA | 34.3000 | South | 0 |
| 1833 | MB | GA | 34.3000 | South | 1 |
| 1834 | MB | GA | 34.3000 | South | 0 |
| 1839 | MB | GA | 34.3000 | South | 0 |
| 1841 | MB | GA | 34.3000 | South | 0 |
| 1842 | MB | GA | 34.3000 | South | 0 |
| 1836 | MB | GA | 34.3000 | South | 1 |
| 1837 | MB | GA | 34.3000 | South | 1 |
| 1830 | MB | GA | 34.3000 | South | 0 |
| 1829 | MB | GA | 34.3000 | South | 0 |
| 1844 | MB | GA | 34.3000 | South | 0 |
| 1845 | MB | GA | 34.3000 | South | 0 |
| 1817 | MB | GA | 34.3000 | South | 1 |
| 1825 | MB | GA | 34.3000 | South | 1 |
| 1835 | MB | GA | 34.3000 | South | 0 |
| 1840 | MB | GA | 34.3000 | South | 0 |
| 1832 | MB | GA | 34.3000 | South | 0 |
| 1838 | MB | GA | 34.3000 | South | 1 |
| 1826 | MB | GA | 34.3000 | South | 0 |
| 1827 | MB | GA | 34.3000 | South | 0 |
| 1828 | MB | GA | 34.3000 | South | 0 |
| 1831 | MB | GA | 34.3000 | South | 0 |
| 1843 | MB | GA | 34.3000 | South | 0 |
| 1846 | MB | GA | 34.3000 | South | 0 |
| 1849 | MBC | GA | 31.5000 | South | 0 |
| 1848 | MBC | GA | 31.5000 | South | 0 |
| 1851 | MBC | GA | 31.5000 | South | 0 |
| 1853 | MBC | GA | 31.5000 | South | 0 |
| 1850 | MBC | GA | 31.5000 | South | 0 |
| 1852 | MBC | GA | 31.5000 | South | 0 |
| 1854 | MBC | GA | 31.5000 | South | 0 |
| 1337 | MT | MN | 44.8000 | North | 0 |
| 1338 | MT | MN | 44.8000 | North | 0 |
| 1339 | MT | MN | 44.8000 | North | 0 |
| 955 | MB | NJ | 39.9300 | Central | 0 |
| 2012 | MB | NJ | 39.9300 | Central | 1 |
| 1761 | MB | NJ | 39.9300 | Central | 0 |
| 1766 | MB | NJ | 39.9300 | Central | 0 |
| 1767 | MB | NJ | 39.9300 | Central | 0 |
| 1774 | MB | NJ | 39.9300 | Central | 0 |
| 1775 | MB | NJ | 39.9300 | Central | 0 |
| 1776 | MB | NJ | 39.9300 | Central | 0 |
| 1777 | MB | NJ | 39.9300 | Central | 0 |
| 1779 | MB | NJ | 39.9300 | Central | 0 |
| 1781 | MB | NJ | 39.9300 | Central | 0 |
| 1783 | MB | NJ | 39.9300 | Central | 0 |
| 1784 | MB | NJ | 39.9300 | Central | 0 |
| 1788 | MB | NJ | 39.9300 | Central | 0 |
| 1789 | MB | NJ | 39.9300 | Central | 0 |
| 1790 | MB | NJ | 39.9300 | Central | 0 |
| 1792 | MB | NJ | 39.9300 | Central | 0 |
| 1793 | MB | NJ | 39.9300 | Central | 0 |
| 1795 | MB | NJ | 39.9300 | Central | 0 |
| 1796 | MB | NJ | 39.9300 | Central | 0 |
| 1797 | MB | NJ | 39.9300 | Central | 0 |
| 1798 | MB | NJ | 39.9300 | Central | 0 |
| 910 | MB | NJ | 39.9300 | Central | 0 |
| 911 | MB | NJ | 39.9300 | Central | 0 |
| 1800 | MB | NJ | 39.9300 | Central | 0 |
| 1804 | MB | NJ | 39.9300 | Central | 0 |
| 1805 | MB | NJ | 39.9300 | Central | 0 |
| 1808 | MB | NJ | 39.9300 | Central | 0 |
| 1802 | MB | NJ | 39.9300 | Central | 0 |
| 1810 | MB | NJ | 39.9300 | Central | 0 |
| 1812 | MB | NJ | 39.9300 | Central | 0 |
| 1813 | MB | NJ | 39.9300 | Central | 0 |
| 1814 | MB | NJ | 39.9300 | Central | 0 |
| 915 | MB | NJ | 39.9300 | Central | 0 |
| 922 | MB | NJ | 39.9300 | Central | 0 |
| 923 | MB | NJ | 39.9300 | Central | 0 |
| 927 | MB | NJ | 39.9300 | Central | 0 |
| 926 | MB | NJ | 39.9300 | Central | 0 |
| 929 | MB | NJ | 39.9300 | Central | 1 |
| 934 | MB | NJ | 39.9300 | Central | 0 |
| 935 | MB | NJ | 39.9300 | Central | 0 |
| 937 | MB | NJ | 39.9300 | Central | 0 |
| 938 | MB | NJ | 39.9300 | Central | 0 |
| 940 | MB | NJ | 39.9300 | Central | 0 |
| 936 | MB | NJ | 39.9300 | Central | 1 |
| 943 | MB | NJ | 39.9300 | Central | 1 |
| 949 | MB | NJ | 39.9300 | Central | 0 |
| 952 | MB | NJ | 39.9300 | Central | 0 |
| 953 | MB | NJ | 39.9300 | Central | 0 |
| 965 | MB | NJ | 39.9300 | Central | 0 |
| 968 | MB | NJ | 39.9300 | Central | 0 |
| 969 | MB | NJ | 39.9300 | Central | 0 |
| 950 | MB | NJ | 39.9300 | Central | 0 |
| 957 | MB | NJ | 39.9300 | Central | 0 |
| 960 | MB | NJ | 39.9300 | Central | 0 |
| 966 | MB | NJ | 39.9300 | Central | 0 |
| 1962 | MB | NJ | 39.9300 | Central | 0 |
| 958 | MB | NJ | 39.9300 | Central | 0 |
| 970 | MB | NJ | 39.9300 | Central | 0 |
| 971 | MB | NJ | 39.9300 | Central | 0 |
| 946 | MB | NJ | 39.9300 | Central | 1 |
| 948 | MB | NJ | 39.9300 | Central | 1 |
| 956 | MB | NJ | 39.9300 | Central | 1 |
| 972 | MB | NJ | 39.9300 | Central | 1 |
| 973 | MB | NJ | 39.9300 | Central | 1 |
| 975 | MB | NJ | 39.9300 | Central | 1 |
| 979 | MB | NJ | 39.9300 | Central | 1 |
| 980 | MB | NJ | 39.9300 | Central | 1 |
| 944 | MB | NJ | 39.9300 | Central | 1 |
| 977 | MB | NJ | 39.9300 | Central | 1 |
| 1964 | MB | NJ | 39.9300 | Central | 0 |
| 1965 | MB | NJ | 39.9300 | Central | 0 |
| 1972 | MB | NJ | 39.9300 | Central | 0 |
| 1978 | MB | NJ | 39.9300 | Central | 0 |
| 1979 | MB | NJ | 39.9300 | Central | 0 |
| 1981 | MB | NJ | 39.9300 | Central | 0 |
| 1994 | MB | NJ | 39.9300 | Central | 0 |
| 1998 | MB | NJ | 39.9300 | Central | 0 |
| 2007 | MB | NJ | 39.9300 | Central | 0 |
| 1966 | MB | NJ | 39.9300 | Central | 0 |
| 1968 | MB | NJ | 39.9300 | Central | 0 |
| 1977 | MB | NJ | 39.9300 | Central | 0 |
| 1983 | MB | NJ | 39.9300 | Central | 0 |
| 1989 | MB | NJ | 39.9300 | Central | 0 |
| 1967 | MB | NJ | 39.9300 | Central | 0 |
| 1990 | MB | NJ | 39.9300 | Central | 0 |
| 2000 | MB | NJ | 39.9300 | Central | 0 |
| 2001 | MB | NJ | 39.9300 | Central | 0 |
| 2003 | MB | NJ | 39.9300 | Central | 0 |
| 2006 | MB | NJ | 39.9300 | Central | 0 |
| 1974 | MB | NJ | 39.9300 | Central | 1 |
| 1988 | MB | NJ | 39.9300 | Central | 1 |
| 1992 | MB | NJ | 39.9300 | Central | 1 |
| 1993 | MB | NJ | 39.9300 | Central | 1 |
| 1996 | MB | NJ | 39.9300 | Central | 1 |
| 1997 | MB | NJ | 39.9300 | Central | 1 |
| 2002 | MB | NJ | 39.9300 | Central | 1 |
| 2019 | MB | NJ | 39.9300 | Central | 0 |
| 2015 | MB | NJ | 39.9300 | Central | 0 |
| 2029 | MB | NJ | 39.9300 | Central | 0 |
| 2013 | MB | NJ | 39.9300 | Central | 1 |
| 2014 | MB | NJ | 39.9300 | Central | 1 |
| 2023 | MB | NJ | 39.9300 | Central | 1 |
| 2027 | MB | NJ | 39.9300 | Central | 1 |
| 2041 | MB | NJ | 39.9300 | Central | 0 |
| 2046 | MB | NJ | 39.9300 | Central | 0 |
| 2055 | MB | NJ | 39.9300 | Central | 0 |
| 2058 | MB | NJ | 39.9300 | Central | 0 |
| 2035 | MB | NJ | 39.9300 | Central | 0 |
| 2051 | MB | NJ | 39.9300 | Central | 0 |
| 2036 | MB | NJ | 39.9300 | Central | 0 |
| 2043 | MB | NJ | 39.9300 | Central | 0 |
| 2050 | MB | NJ | 39.9300 | Central | 0 |
| 2052 | MB | NJ | 39.9300 | Central | 0 |
| 2053 | MB | NJ | 39.9300 | Central | 0 |
| 2037 | MB | NJ | 39.9300 | Central | 1 |
| 2038 | MB | NJ | 39.9300 | Central | 1 |
| 2044 | MB | NJ | 39.9300 | Central | 1 |
| 2045 | MB | NJ | 39.9300 | Central | 1 |
| 2049 | MB | NJ | 39.9300 | Central | 1 |
| 2056 | MB | NJ | 39.9300 | Central | 1 |
| 2060 | MB | NJ | 39.9300 | Central | 0 |
| 2066 | MB | NJ | 39.9300 | Central | 0 |
| 2068 | MB | NJ | 39.9300 | Central | 0 |
| 2069 | MB | NJ | 39.9300 | Central | 0 |
| 2072 | MB | NJ | 39.9300 | Central | 0 |
| 2067 | MB | NJ | 39.9300 | Central | 0 |
| 2064 | MB | NJ | 39.9300 | Central | 0 |
| 2065 | MB | NJ | 39.9300 | Central | 1 |
| 2075 | MB | NJ | 39.9300 | Central | 1 |
| 1050 | MB | NJ | 39.9300 | Central | 0 |
| 1053 | MB | NJ | 39.9300 | Central | 0 |
| 1054 | MB | NJ | 39.9300 | Central | 0 |
| 1055 | MB | NJ | 39.9300 | Central | 0 |
| 1058 | MB | NJ | 39.9300 | Central | 0 |
| 1061 | MB | NJ | 39.9300 | Central | 0 |
| 1048 | MB | NJ | 39.9300 | Central | 0 |
| 1052 | MB | NJ | 39.9300 | Central | 0 |
| 1044 | MB | NJ | 39.9300 | Central | 0 |
| 1046 | MB | NJ | 39.9300 | Central | 0 |
| 1047 | MB | NJ | 39.9300 | Central | 0 |
| 1056 | MB | NJ | 39.9300 | Central | 0 |
| 1063 | MB | NJ | 39.9300 | Central | 0 |
| 1064 | MB | NJ | 39.9300 | Central | 0 |
| 1045 | MB | NJ | 39.9300 | Central | 1 |
| 1049 | MB | NJ | 39.9300 | Central | 1 |
| 1051 | MB | NJ | 39.9300 | Central | 1 |
| 1057 | MB | NJ | 39.9300 | Central | 1 |
| 1059 | MB | NJ | 39.9300 | Central | 1 |
| 1060 | MB | NJ | 39.9300 | Central | 1 |
| 1062 | MB | NJ | 39.9300 | Central | 1 |
| 1760 | MB | NJ | 39.9300 | Central | 0 |
| 1762 | MB | NJ | 39.9300 | Central | 0 |
| 1763 | MB | NJ | 39.9300 | Central | 0 |
| 1764 | MB | NJ | 39.9300 | Central | 0 |
| 1765 | MB | NJ | 39.9300 | Central | 0 |
| 1768 | MB | NJ | 39.9300 | Central | 0 |
| 1769 | MB | NJ | 39.9300 | Central | 0 |
| 1770 | MB | NJ | 39.9300 | Central | 0 |
| 1771 | MB | NJ | 39.9300 | Central | 0 |
| 1772 | MB | NJ | 39.9300 | Central | 0 |
| 1773 | MB | NJ | 39.9300 | Central | 0 |
| 1778 | MB | NJ | 39.9300 | Central | 0 |
| 1780 | MB | NJ | 39.9300 | Central | 0 |
| 1782 | MB | NJ | 39.9300 | Central | 0 |
| 1785 | MB | NJ | 39.9300 | Central | 0 |
| 1786 | MB | NJ | 39.9300 | Central | 0 |
| 1787 | MB | NJ | 39.9300 | Central | 0 |
| 1791 | MB | NJ | 39.9300 | Central | 0 |
| 1794 | MB | NJ | 39.9300 | Central | 1 |
| 912 | MB | NJ | 39.9300 | Central | 0 |
| 913 | MB | NJ | 39.9300 | Central | 0 |
| 1799 | MB | NJ | 39.9300 | Central | 0 |
| 1806 | MB | NJ | 39.9300 | Central | 0 |
| 1807 | MB | NJ | 39.9300 | Central | 0 |
| 1809 | MB | NJ | 39.9300 | Central | 0 |
| 1816 | MB | NJ | 39.9300 | Central | 0 |
| 1811 | MB | NJ | 39.9300 | Central | 0 |
| 1815 | MB | NJ | 39.9300 | Central | 0 |
| 914 | MB | NJ | 39.9300 | Central | 1 |
| 1801 | MB | NJ | 39.9300 | Central | 1 |
| 1803 | MB | NJ | 39.9300 | Central | 1 |
| 916 | MB | NJ | 39.9300 | Central | 0 |
| 918 | MB | NJ | 39.9300 | Central | 0 |
| 919 | MB | NJ | 39.9300 | Central | 0 |
| 925 | MB | NJ | 39.9300 | Central | 0 |
| 928 | MB | NJ | 39.9300 | Central | 0 |
| 930 | MB | NJ | 39.9300 | Central | 0 |
| 932 | MB | NJ | 39.9300 | Central | 0 |
| 933 | MB | NJ | 39.9300 | Central | 0 |
| 920 | MB | NJ | 39.9300 | Central | 0 |
| 921 | MB | NJ | 39.9300 | Central | 0 |
| 917 | MB | NJ | 39.9300 | Central | 0 |
| 924 | MB | NJ | 39.9300 | Central | 1 |
| 931 | MB | NJ | 39.9300 | Central | 1 |
| 939 | MB | NJ | 39.9300 | Central | 0 |
| 941 | MB | NJ | 39.9300 | Central | 0 |
| 942 | MB | NJ | 39.9300 | Central | 0 |
| 945 | MB | NJ | 39.9300 | Central | 0 |
| 961 | MB | NJ | 39.9300 | Central | 0 |
| 963 | MB | NJ | 39.9300 | Central | 0 |
| 967 | MB | NJ | 39.9300 | Central | 0 |
| 954 | MB | NJ | 39.9300 | Central | 0 |
| 959 | MB | NJ | 39.9300 | Central | 0 |
| 962 | MB | NJ | 39.9300 | Central | 0 |
| 947 | MB | NJ | 39.9300 | Central | 0 |
| 964 | MB | NJ | 39.9300 | Central | 0 |
| 1963 | MB | NJ | 39.9300 | Central | 0 |
| 951 | MB | NJ | 39.9300 | Central | 1 |
| 974 | MB | NJ | 39.9300 | Central | 1 |
| 976 | MB | NJ | 39.9300 | Central | 1 |
| 978 | MB | NJ | 39.9300 | Central | 1 |
| 1969 | MB | NJ | 39.9300 | Central | 0 |
| 1970 | MB | NJ | 39.9300 | Central | 0 |
| 1985 | MB | NJ | 39.9300 | Central | 0 |
| 1987 | MB | NJ | 39.9300 | Central | 0 |
| 1995 | MB | NJ | 39.9300 | Central | 0 |
| 2004 | MB | NJ | 39.9300 | Central | 0 |
| 2005 | MB | NJ | 39.9300 | Central | 0 |
| 2008 | MB | NJ | 39.9300 | Central | 0 |
| 2009 | MB | NJ | 39.9300 | Central | 0 |
| 2010 | MB | NJ | 39.9300 | Central | 0 |
| 2011 | MB | NJ | 39.9300 | Central | 0 |
| 1971 | MB | NJ | 39.9300 | Central | 0 |
| 1980 | MB | NJ | 39.9300 | Central | 0 |
| 1984 | MB | NJ | 39.9300 | Central | 0 |
| 1991 | MB | NJ | 39.9300 | Central | 0 |
| 1999 | MB | NJ | 39.9300 | Central | 0 |
| 1975 | MB | NJ | 39.9300 | Central | 1 |
| 1976 | MB | NJ | 39.9300 | Central | 1 |
| 1982 | MB | NJ | 39.9300 | Central | 1 |
| 1986 | MB | NJ | 39.9300 | Central | 1 |
| 2016 | MB | NJ | 39.9300 | Central | 0 |
| 2020 | MB | NJ | 39.9300 | Central | 0 |
| 2021 | MB | NJ | 39.9300 | Central | 0 |
| 2028 | MB | NJ | 39.9300 | Central | 0 |
| 2030 | MB | NJ | 39.9300 | Central | 0 |
| 2031 | MB | NJ | 39.9300 | Central | 0 |
| 2018 | MB | NJ | 39.9300 | Central | 0 |
| 2024 | MB | NJ | 39.9300 | Central | 0 |
| 2017 | MB | NJ | 39.9300 | Central | 1 |
| 2022 | MB | NJ | 39.9300 | Central | 1 |
| 2025 | MB | NJ | 39.9300 | Central | 1 |
| 2026 | MB | NJ | 39.9300 | Central | 1 |
| 2033 | MB | NJ | 39.9300 | Central | 0 |
| 2032 | MB | NJ | 39.9300 | Central | 1 |
| 2034 | MB | NJ | 39.9300 | Central | 0 |
| 2054 | MB | NJ | 39.9300 | Central | 0 |
| 2057 | MB | NJ | 39.9300 | Central | 0 |
| 2042 | MB | NJ | 39.9300 | Central | 0 |
| 2039 | MB | NJ | 39.9300 | Central | 1 |
| 2040 | MB | NJ | 39.9300 | Central | 1 |
| 2047 | MB | NJ | 39.9300 | Central | 1 |
| 2048 | MB | NJ | 39.9300 | Central | 1 |
| 2059 | MB | NJ | 39.9300 | Central | 1 |
| 2062 | MB | NJ | 39.9300 | Central | 0 |
| 2070 | MB | NJ | 39.9300 | Central | 0 |
| 2063 | MB | NJ | 39.9300 | Central | 0 |
| 2073 | MB | NJ | 39.9300 | Central | 0 |
| 2071 | MB | NJ | 39.9300 | Central | 0 |
| 2061 | MB | NJ | 39.9300 | Central | 1 |
| 2074 | MB | NJ | 39.9300 | Central | 1 |
| 2076 | MB | NJ | 39.9300 | Central | 1 |
| 2077 | MB | NJ | 39.9300 | Central | 1 |
| 1018 | MB | NJ | 39.9300 | Central | 0 |
| 1036 | MB | NJ | 39.9300 | Central | 0 |
| 1024 | MB | NJ | 39.9300 | Central | 0 |
| 1025 | MB | NJ | 39.9300 | Central | 0 |
| 1026 | MB | NJ | 39.9300 | Central | 0 |
| 1027 | MB | NJ | 39.9300 | Central | 0 |
| 1031 | MB | NJ | 39.9300 | Central | 0 |
| 1038 | MB | NJ | 39.9300 | Central | 0 |
| 1039 | MB | NJ | 39.9300 | Central | 0 |
| 1041 | MB | NJ | 39.9300 | Central | 0 |
| 1017 | MB | NJ | 39.9300 | Central | 0 |
| 1021 | MB | NJ | 39.9300 | Central | 0 |
| 1033 | MB | NJ | 39.9300 | Central | 0 |
| 1034 | MB | NJ | 39.9300 | Central | 0 |
| 1040 | MB | NJ | 39.9300 | Central | 0 |
| 1019 | MB | NJ | 39.9300 | Central | 1 |
| 1020 | MB | NJ | 39.9300 | Central | 1 |
| 1022 | MB | NJ | 39.9300 | Central | 1 |
| 1023 | MB | NJ | 39.9300 | Central | 1 |
| 1028 | MB | NJ | 39.9300 | Central | 1 |
| 1029 | MB | NJ | 39.9300 | Central | 1 |
| 1030 | MB | NJ | 39.9300 | Central | 1 |
| 1032 | MB | NJ | 39.9300 | Central | 1 |
| 1035 | MB | NJ | 39.9300 | Central | 1 |
| 1037 | MB | NJ | 39.9300 | Central | 1 |
| 1042 | MB | NJ | 39.9300 | Central | 1 |
| 1043 | MB | NJ | 39.9300 | Central | 1 |
| 1126 | MO | IA | 42.1000 | North | 0 |
| 1114 | MO | IA | 42.1000 | North | 0 |
| 1119 | MO | IA | 42.1000 | North | 0 |
| 1120 | MO | IA | 42.1000 | North | 0 |
| 1124 | MO | IA | 42.1000 | North | 0 |
| 1125 | MO | IA | 42.1000 | North | 0 |
| 1128 | MO | IA | 42.1000 | North | 0 |
| 1129 | MO | IA | 42.1000 | North | 0 |
| 1130 | MO | IA | 42.1000 | North | 0 |
| 1115 | MO | IA | 42.1000 | North | 0 |
| 1116 | MO | IA | 42.1000 | North | 0 |
| 1117 | MO | IA | 42.1000 | North | 0 |
| 1118 | MO | IA | 42.1000 | North | 0 |
| 1121 | MO | IA | 42.1000 | North | 0 |
| 1122 | MO | IA | 42.1000 | North | 0 |
| 1123 | MO | IA | 42.1000 | North | 0 |
| 1127 | MO | IA | 42.1000 | North | 0 |
| 1606 | ML | IL | 42.0400 | North | 0 |
| 1605 | ML | IL | 42.0400 | North | 0 |
| 1609 | ML | IL | 42.0400 | North | 0 |
| 1607 | ML | IL | 42.0400 | North | 1 |
| 1608 | ML | IL | 42.0400 | North | 0 |
| 697 | OM | GA | 32.4000 | South | 0 |
| 687 | OM | GA | 32.4000 | South | 0 |
| 689 | OM | GA | 32.4000 | South | 0 |
| 690 | OM | GA | 32.4000 | South | 0 |
| 688 | OM | GA | 32.4000 | South | 1 |
| 691 | OM | GA | 32.4000 | South | 0 |
| 692 | OM | GA | 32.4000 | South | 0 |
| 694 | OM | GA | 32.4000 | South | 0 |
| 696 | OM | GA | 32.4000 | South | 0 |
| 695 | OM | GA | 32.4000 | South | 1 |
| 698 | OM | GA | 32.4000 | South | 0 |
| 679 | OM | GA | 32.4000 | South | 0 |
| 680 | OM | GA | 32.4000 | South | 0 |
| 681 | OM | GA | 32.4000 | South | 0 |
| 682 | OM | GA | 32.4000 | South | 0 |
| 683 | OM | GA | 32.4000 | South | 0 |
| 684 | OM | GA | 32.4000 | South | 0 |
| 686 | OM | GA | 32.4000 | South | 0 |
| 693 | OM | GA | 32.4000 | South | 0 |
| 685 | OM | GA | 32.4000 | South | 0 |
| 1204 | PS | MI | 41.9000 | North | 0 |
| 1205 | PS | MI | 41.9000 | North | 1 |
| 1206 | PS | MI | 41.9000 | North | 1 |
| 1200 | PS | MI | 41.9000 | North | 0 |
| 1203 | PS | MI | 41.9000 | North | 0 |
| 1199 | PS | MI | 41.9000 | North | 0 |
| 1201 | PS | MI | 41.9000 | North | 0 |
| 1202 | PS | MI | 41.9000 | North | 0 |
| 648 | PC | MN | 44.8000 | North | 0 |
| 656 | PC | MN | 45.0000 | North | 0 |
| 660 | PC | MN | 45.0000 | North | 0 |
| 652 | PC | MN | 45.0000 | North | 0 |
| 654 | PC | MN | 45.0000 | North | 0 |
| 649 | PC | MN | 45.0000 | North | 0 |
| 651 | PC | MN | 45.0000 | North | 0 |
| 653 | PC | MN | 45.0000 | North | 0 |
| 657 | PC | MN | 45.0000 | North | 0 |
| 658 | PC | MN | 45.0000 | North | 0 |
| 659 | PC | MN | 45.0000 | North | 0 |
| 661 | PC | MN | 45.0000 | North | 0 |
| 650 | PC | MN | 45.0000 | North | 1 |
| 655 | PC | MN | 45.0000 | North | 1 |
| 662 | PC | MN | 45.0000 | North | 0 |
| 1327 | PB | NY | 41.2000 | Central | 0 |
| 1332 | PB | NY | 41.2000 | Central | 0 |
| 1328 | PB | NY | 41.2000 | Central | 0 |
| 1329 | PB | NY | 41.2000 | Central | 0 |
| 1330 | PB | NY | 41.2000 | Central | 0 |
| 1331 | PB | NY | 41.2000 | Central | 0 |
| 1324 | PB | NY | 41.2000 | Central | 0 |
| 1326 | PB | NY | 41.2000 | Central | 0 |
| 1261 | PJP | MN | 45.3000 | North | 0 |
| 1262 | PJP | MN | 45.3000 | North | 0 |
| 1263 | PJP | MN | 45.3000 | North | 0 |
| 1264 | PJP | MN | 45.3000 | North | 0 |
| 1260 | PJP | MN | 45.3000 | North | 0 |
| 1265 | PJP | MN | 45.3000 | North | 0 |
| 1185 | RP | GA | 34.3000 | South | 0 |
| 1179 | RP | GA | 34.3000 | South | 0 |
| 1180 | RP | GA | 34.3000 | South | 0 |
| 1182 | RP | GA | 34.3000 | South | 0 |
| 1186 | RP | GA | 34.3000 | South | 0 |
| 1187 | RP | GA | 34.3000 | South | 0 |
| 1189 | RP | GA | 34.3000 | South | 0 |
| 1191 | RP | GA | 34.3000 | South | 0 |
| 1193 | RP | GA | 34.3000 | South | 0 |
| 1183 | RP | GA | 34.3000 | South | 0 |
| 1184 | RP | GA | 34.3000 | South | 0 |
| 1190 | RP | GA | 34.3000 | South | 0 |
| 1181 | RP | GA | 34.3000 | South | 0 |
| 1188 | RP | GA | 34.3000 | South | 1 |
| 1192 | RP | GA | 34.3000 | South | 1 |
| 1194 | RP | GA | 34.3000 | South | 0 |
| 1195 | RP | GA | 34.3000 | South | 0 |
| 1196 | RP | GA | 34.3000 | South | 0 |
| 1197 | RP | GA | 34.3000 | South | 0 |
| 664 | RS | KS | 38.5700 | Central | 0 |
| 665 | RS | KS | 38.5700 | Central | 0 |
| 666 | RS | KS | 38.5700 | Central | 0 |
| 663 | RS | KS | 38.5700 | Central | 0 |
| 1636 | SQ | MI | 41.9000 | North | 0 |
| 1706 | SC | IA | 42.6000 | North | 1 |
| 1722 | SC | IA | 42.6000 | North | 1 |
| 1707 | SC | IA | 42.6000 | North | 0 |
| 1719 | SC | IA | 42.6000 | North | 0 |
| 1720 | SC | IA | 42.6000 | North | 0 |
| 1718 | SC | IA | 42.6000 | North | 0 |
| 1710 | SC | IA | 42.6000 | North | 0 |
| 1711 | SC | IA | 42.6000 | North | 0 |
| 1708 | SC | IA | 42.6000 | North | 0 |
| 1709 | SC | IA | 42.6000 | North | 0 |
| 1712 | SC | IA | 42.6000 | North | 1 |
| 1716 | SC | IA | 42.6000 | North | 0 |
| 1713 | SC | IA | 42.6000 | North | 1 |
| 1714 | SC | IA | 42.6000 | North | 1 |
| 1717 | SC | IA | 42.6000 | North | 0 |
| 1715 | SC | IA | 42.6000 | North | 1 |
| 1721 | SC | IA | 42.6000 | North | 1 |
| 1008 | SS | IL | 42.0400 | North | 0 |
| 1009 | SS | IL | 42.0400 | North | 0 |
| 1010 | SS | IL | 42.0400 | North | 0 |
| 835 | SD | WI | 44.3000 | North | 0 |
| 831 | SD | WI | 44.3000 | North | 0 |
| 832 | SD | WI | 44.3000 | North | 0 |
| 833 | SD | WI | 44.3000 | North | 0 |
| 836 | SD | WI | 44.3000 | North | 0 |
| 834 | SD | WI | 44.3000 | North | 1 |
| 837 | SD | WI | 44.3000 | North | 0 |
| 838 | SD | WI | 44.3000 | North | 0 |
| 839 | SD | WI | 44.3000 | North | 0 |
| 807 | SD | WI | 44.3000 | North | 0 |
| 815 | SD | WI | 44.3000 | North | 0 |
| 810 | SD | WI | 44.3000 | North | 0 |
| 814 | SD | WI | 44.3000 | North | 0 |
| 811 | SD | WI | 44.3000 | North | 0 |
| 796 | SD | WI | 44.3000 | North | 0 |
| 797 | SD | WI | 44.3000 | North | 0 |
| 798 | SD | WI | 44.3000 | North | 0 |
| 821 | SD | WI | 44.3000 | North | 0 |
| 822 | SD | WI | 44.3000 | North | 0 |
| 823 | SD | WI | 44.3000 | North | 0 |
| 824 | SD | WI | 44.3000 | North | 0 |
| 825 | SD | WI | 44.3000 | North | 0 |
| 826 | SD | WI | 44.3000 | North | 0 |
| 827 | SD | WI | 44.3000 | North | 0 |
| 812 | SD | WI | 44.3000 | North | 0 |
| 813 | SD | WI | 44.3000 | North | 0 |
| 816 | SD | WI | 44.3000 | North | 0 |
| 817 | SD | WI | 44.3000 | North | 0 |
| 818 | SD | WI | 44.3000 | North | 0 |
| 820 | SD | WI | 44.3000 | North | 0 |
| 819 | SD | WI | 44.3000 | North | 0 |
| 794 | SD | WI | 44.3000 | North | 0 |
| 795 | SD | WI | 44.3000 | North | 0 |
| 793 | SD | WI | 44.3000 | North | 1 |
| 801 | SD | WI | 44.3000 | North | 1 |
| 787 | SD | WI | 44.3000 | North | 0 |
| 799 | SD | WI | 44.3000 | North | 0 |
| 800 | SD | WI | 44.3000 | North | 0 |
| 802 | SD | WI | 44.3000 | North | 0 |
| 803 | SD | WI | 44.3000 | North | 0 |
| 789 | SD | WI | 44.3000 | North | 0 |
| 790 | SD | WI | 44.3000 | North | 0 |
| 791 | SD | WI | 44.3000 | North | 0 |
| 792 | SD | WI | 44.3000 | North | 1 |
| 804 | SD | WI | 44.3000 | North | 0 |
| 805 | SD | WI | 44.3000 | North | 0 |
| 806 | SD | WI | 44.3000 | North | 0 |
| 808 | SD | WI | 44.3000 | North | 0 |
| 809 | SD | WI | 44.3000 | North | 0 |
| 1361 | SM | GA | 33.6000 | South | 0 |
| 1370 | SM | GA | 33.6000 | South | 0 |
| 1357 | SM | GA | 33.6000 | South | 0 |
| 1358 | SM | GA | 33.6000 | South | 0 |
| 1359 | SM | GA | 33.6000 | South | 0 |
| 1360 | SM | GA | 33.6000 | South | 0 |
| 1364 | SM | GA | 33.6000 | South | 0 |
| 1362 | SM | GA | 33.6000 | South | 0 |
| 1363 | SM | GA | 33.6000 | South | 0 |
| 1366 | SM | GA | 33.6000 | South | 0 |
| 1368 | SM | GA | 33.6000 | South | 0 |
| 1372 | SM | GA | 33.6000 | South | 0 |
| 1356 | SM | GA | 33.6000 | South | 1 |
| 1365 | SM | GA | 33.6000 | South | 0 |
| 1369 | SM | GA | 33.6000 | South | 0 |
| 1371 | SM | GA | 33.6000 | South | 0 |
| 1367 | SM | GA | 33.6000 | South | 0 |
| 1373 | SM | GA | 33.6000 | South | 0 |
| 1375 | SM | GA | 33.6000 | South | 0 |
| 1374 | SM | GA | 33.6000 | South | 1 |
| 1879 | SH | VT | 43.3000 | North | 0 |
| 593 | SH | VT | 43.3000 | North | 0 |
| 595 | SH | VT | 43.3000 | North | 0 |
| 1866 | SH | VT | 43.3000 | North | 0 |
| 1868 | SH | VT | 43.3000 | North | 0 |
| 1869 | SH | VT | 43.3000 | North | 0 |
| 1870 | SH | VT | 43.3000 | North | 0 |
| 1875 | SH | VT | 43.3000 | North | 0 |
| 1878 | SH | VT | 43.3000 | North | 0 |
| 1880 | SH | VT | 43.3000 | North | 0 |
| 1881 | SH | VT | 43.3000 | North | 0 |
| 1884 | SH | VT | 43.3000 | North | 0 |
| 1882 | SH | VT | 43.3000 | North | 0 |
| 1885 | SH | VT | 43.3000 | North | 0 |
| 1886 | SH | VT | 43.3000 | North | 0 |
| 1873 | SH | VT | 43.3000 | North | 0 |
| 1874 | SH | VT | 43.3000 | North | 0 |
| 1887 | SH | VT | 43.3000 | North | 0 |
| 1896 | SH | VT | 43.3000 | North | 0 |
| 1894 | SH | VT | 43.3000 | North | 0 |
| 1890 | SH | VT | 43.3000 | North | 0 |
| 1901 | SH | VT | 43.3000 | North | 0 |
| 1898 | SH | VT | 43.3000 | North | 0 |
| 1899 | SH | VT | 43.3000 | North | 0 |
| 1900 | SH | VT | 43.3000 | North | 0 |
| 1904 | SH | VT | 43.3000 | North | 0 |
| 1905 | SH | VT | 43.3000 | North | 0 |
| 1906 | SH | VT | 43.3000 | North | 0 |
| 592 | SH | VT | 43.3000 | North | 0 |
| 1865 | SH | VT | 43.3000 | North | 0 |
| 1867 | SH | VT | 43.3000 | North | 0 |
| 1871 | SH | VT | 43.3000 | North | 0 |
| 1872 | SH | VT | 43.3000 | North | 0 |
| 1876 | SH | VT | 43.3000 | North | 0 |
| 1883 | SH | VT | 43.3000 | North | 0 |
| 1877 | SH | VT | 43.3000 | North | 1 |
| 1888 | SH | VT | 43.3000 | North | 0 |
| 1889 | SH | VT | 43.3000 | North | 0 |
| 1892 | SH | VT | 43.3000 | North | 0 |
| 1893 | SH | VT | 43.3000 | North | 0 |
| 1895 | SH | VT | 43.3000 | North | 0 |
| 1891 | SH | VT | 43.3000 | North | 0 |
| 1897 | SH | VT | 43.3000 | North | 0 |
| 1903 | SH | VT | 43.3000 | North | 0 |
| 1902 | SH | VT | 43.3000 | North | 0 |
| 590 | SH | VT | 43.3000 | North | 0 |
| 591 | SH | VT | 43.3000 | North | 0 |
| 1267 | SB | IN | 40.3000 | Central | 0 |
| 1266 | SB | IN | 40.3000 | Central | 0 |
| 1268 | SB | IN | 40.3000 | Central | 0 |
| 1344 | SC | VA | 36.9800 | Central | 1 |
| 1345 | SC | VA | 36.9800 | Central | 0 |
| 1351 | SC | VA | 36.9800 | Central | 0 |
| 1352 | SC | VA | 36.9800 | Central | 1 |
| 1346 | SC | VA | 36.9800 | Central | 1 |
| 1340 | SC | VA | 36.9800 | Central | 0 |
| 1349 | SC | VA | 36.9800 | Central | 0 |
| 1350 | SC | VA | 36.9800 | Central | 1 |
| 1348 | SC | VA | 36.9800 | Central | 0 |
| 1353 | SC | VA | 36.9800 | Central | 1 |
| 1355 | SC | VA | 36.9800 | Central | 0 |
| 1354 | SC | VA | 36.9800 | Central | 1 |
| 700 | TW | OH | 40.3900 | Central | 0 |
| 701 | TW | OH | 40.3900 | Central | 1 |
| 703 | TW | OH | 40.3900 | Central | 1 |
| 706 | TW | OH | 40.3900 | Central | 0 |
| 710 | TW | OH | 40.3900 | Central | 0 |
| 711 | TW | OH | 40.3900 | Central | 0 |
| 713 | TW | OH | 40.3900 | Central | 0 |
| 714 | TW | OH | 40.3900 | Central | 0 |
| 717 | TW | OH | 40.3900 | Central | 0 |
| 723 | TW | OH | 40.3900 | Central | 0 |
| 728 | TW | OH | 40.3900 | Central | 0 |
| 731 | TW | OH | 40.3900 | Central | 0 |
| 727 | TW | OH | 40.3900 | Central | 0 |
| 734 | TW | OH | 40.3900 | Central | 0 |
| 724 | TW | OH | 40.3900 | Central | 0 |
| 725 | TW | OH | 40.3900 | Central | 1 |
| 726 | TW | OH | 40.3900 | Central | 1 |
| 730 | TW | OH | 40.3900 | Central | 1 |
| 739 | TW | OH | 40.3900 | Central | 0 |
| 739 | TW | OH | 40.3900 | Central | 0 |
| 740 | TW | OH | 40.3900 | Central | 0 |
| 744 | TW | OH | 40.3900 | Central | 0 |
| 747 | TW | OH | 40.3900 | Central | 0 |
| 699 | TW | OH | 40.3900 | Central | 0 |
| 702 | TW | OH | 40.3900 | Central | 1 |
| 704 | TW | OH | 40.3900 | Central | 0 |
| 705 | TW | OH | 40.3900 | Central | 0 |
| 707 | TW | OH | 40.3900 | Central | 0 |
| 708 | TW | OH | 40.3900 | Central | 0 |
| 709 | TW | OH | 40.3900 | Central | 0 |
| 712 | TW | OH | 40.3900 | Central | 1 |
| 716 | TW | OH | 40.3900 | Central | 0 |
| 715 | TW | OH | 40.3900 | Central | 1 |
| 718 | TW | OH | 40.3900 | Central | 1 |
| 720 | TW | OH | 40.3900 | Central | 0 |
| 733 | TW | OH | 40.3900 | Central | 0 |
| 719 | TW | OH | 40.3900 | Central | 0 |
| 721 | TW | OH | 40.3900 | Central | 1 |
| 722 | TW | OH | 40.3900 | Central | 1 |
| 729 | TW | OH | 40.3900 | Central | 1 |
| 732 | TW | OH | 40.3900 | Central | 1 |
| 736 | TW | OH | 40.3900 | Central | 0 |
| 737 | TW | OH | 40.3900 | Central | 0 |
| 741 | TW | OH | 40.3900 | Central | 0 |
| 742 | TW | OH | 40.3900 | Central | 0 |
| 743 | TW | OH | 40.3900 | Central | 0 |
| 745 | TW | OH | 40.3900 | Central | 0 |
| 735 | TW | OH | 40.3900 | Central | 0 |
| 738 | TW | OH | 40.3900 | Central | 1 |
| 746 | TW | OH | 40.3900 | Central | 0 |
| 1140 | TS | GA | 34.3000 | South | 0 |
| 1153 | TS | GA | 34.3000 | South | 0 |
| 1157 | TS | GA | 34.3000 | South | 0 |
| 1151 | TS | GA | 34.3000 | South | 1 |
| 1141 | TS | GA | 34.3000 | South | 0 |
| 1142 | TS | GA | 34.3000 | South | 0 |
| 1143 | TS | GA | 34.3000 | South | 0 |
| 1145 | TS | GA | 34.3000 | South | 0 |
| 1144 | TS | GA | 34.3000 | South | 1 |
| 1146 | TS | GA | 34.3000 | South | 0 |
| 1147 | TS | GA | 34.3000 | South | 0 |
| 1148 | TS | GA | 34.3000 | South | 0 |
| 1149 | TS | GA | 34.3000 | South | 0 |
| 1150 | TS | GA | 34.3000 | South | 0 |
| 1154 | TS | GA | 34.3000 | South | 0 |
| 1156 | TS | GA | 34.3000 | South | 0 |
| 1158 | TS | GA | 34.3000 | South | 0 |
| 1152 | TS | GA | 34.3000 | South | 0 |
| 1155 | TS | GA | 34.3000 | South | 0 |
| 1159 | TS | GA | 34.3000 | South | 0 |
| 607 | TZM | WI | 42.7000 | North | 0 |
| 609 | TZM | WI | 42.7000 | North | 1 |
| 608 | TZM | WI | 42.7000 | North | 0 |
